# Path segmentation for beginners: an overview of current methods for detecting changes in animal movement patterns

**DOI:** 10.1186/s40462-016-0086-5

**Published:** 2016-09-01

**Authors:** Hendrik Edelhoff, Johannes Signer, Niko Balkenhol

**Affiliations:** Department of Wildlife Sciences, University of Göttingen, Büsgenweg 3, 37077 Göttingen, Germany

**Keywords:** Path topology, Telemetry, GPS, Animal behavior, State-space models, Bio-logging, Path segmentation, Path-level analyses

## Abstract

Increased availability of high-resolution movement data has led to the development of numerous methods for studying changes in animal movement behavior. Path segmentation methods provide basics for detecting movement changes and the behavioral mechanisms driving them. However, available path segmentation methods differ vastly with respect to underlying statistical assumptions and output produced. Consequently, it is currently difficult for researchers new to path segmentation to gain an overview of the different methods, and choose one that is appropriate for their data and research questions.

Here, we provide an overview of different methods for segmenting movement paths according to potential changes in underlying behavior. To structure our overview, we outline three broad types of research questions that are commonly addressed through path segmentation: *1)* the quantitative description of movement patterns, *2)* the detection of significant change-points, and *3)* the identification of underlying processes or ‘hidden states’. We discuss advantages and limitations of different approaches for addressing these research questions using path-level movement data, and present general guidelines for choosing methods based on data characteristics and questions. Our overview illustrates the large diversity of available path segmentation approaches, highlights the need for studies that compare the utility of different methods, and identifies opportunities for future developments in path-level data analysis.

**Electronic supplementary material:**

The online version of this article (doi:10.1186/s40462-016-0086-5) contains supplementary material, which is available to authorized users.

## Background

Movement is an important life history trait in organismal ecology. Individual movement decisions and capacities affect habitat-dependent space-use and foraging strategies, as well as dispersal and migration [1, 2]. Changes in movement behavior impact individual fitness, reproductive success and survival [3, 4], ultimately driving population dynamics and evolution of species. The importance of movement has led to the emergence of the movement ecology paradigm, which provides a fundamental conceptual framework for studying movement in a holistic and mechanistic manner [5].

For animals, modern tracking devices (e.g., GPS or ARGOS) make it possible to gather relocation data at increasingly fine spatial and temporal resolutions, thereby providing the data necessary to address comprehensive questions about how individuals perceive, react to, utilize, or even change their environment [6, 7]. Traditionally, animal relocation data were used in different variants of point pattern analyses in order to describe space use and resource selection as well as home ranges and territorial behavior [8–10]. These methods are especially useful when relocations are sampled at low frequencies (e.g., several hours or days) or with large temporal gaps. However, researchers can now collect relocation data for mobile animals at intervals of minutes (e.g., [11]) or even seconds (e.g., [12]). Rather than analyzing such high-frequency data as mere point patterns, they are often treated as movement paths, which provide a temporal sequence of the steps an animal took through space [13]. An important advantage of analyzing animal movements at the path-level is the enhanced opportunity to learn about the behavior driving the observed movement patterns.

Path segmentation methods are perhaps most widely-used for identifying behavioral states from path-level movement data. These methods essentially dissect movement paths into segments that are assumed to reflect different underlying behaviors. By defining behavioral states from the paths and then linking state-dependent movements to the environment, scientists can gain an enhanced understanding of the biological processes influencing the movement behavior of animals [14, 15].

Given the tremendous capabilities of path segmentation for movement ecology, it is not surprising that the number of approaches suggested for segmenting a path and detecting behavioral states is growing rapidly. However, many of these methods have their roots in non-ecological scientific disciplines and gaining a comprehensive understanding of the plethora of available methods can be time-consuming and even frustrating, which likely results in path-level analyses not being used as often and as efficiently as possible.

Here, we offer an overview of available methods for segmenting animal movement paths to detect underlying behavioral states. For this, we first introduce the basics of path-level analyses and relevant terms for distinguishing different movement types. Next, we outline some of the major differences between analytical approaches and suggest general considerations for matching available methods to three broad types of research questions: *1)* the quantitative description of movement patterns, *2)* the detection of significant change-points, or *3)* the identification of underlying processes (“hidden states”). To illustrate our suggestions, we also apply multiple methods to a simulated dataset. We include examples of different ecologically relevant movement processes at varying temporal scales (e.g., diel and annual time scales), as well as behavioral responses to habitat configuration to provide more insight on the application of the presented segmentation approaches. Finally, we discuss remaining challenges and suggest future research avenues for path segmentation. Our overview is specifically intended as a starting point for beginners with little or no experience in path-level analysis of telemetry data, and we therefore avoid statistical details as much as possible. These details can be found in the supplement and also the references given for the individual methods.

## Basics of path-level analyses

### Movement paths and trajectories

**Fig. 1:**
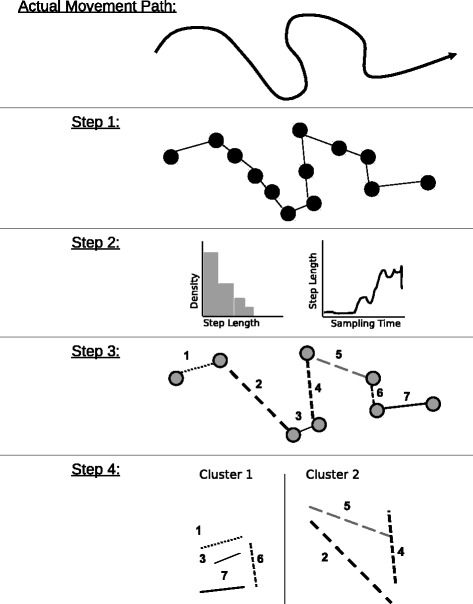
Overview of important steps throughout a segmentation analysis. In general, the actual continuous movement path of an organism is sampled as a set of consecutive relocations (Step 1; e.g., field work). Step 2: exploratory and descriptive analyses of path characteristics exploring and visualizing of the data structure. Step 3: applying one or several path segmentation method(s) to objectively distinguish different movement states. Step 4: Some methods require the use of clustering and summary statistics to quantify differences in distinguished movement states, and to facilitate biological interpretation in terms of behavioral modes

Usually, we cannot observe the complete, continuous movement path of an animal. Instead, we sample a set of discrete relocations to approximate the animals’ actual movement path [16] (Step 1 in Fig. 1). The resulting sequence of consecutive records of the location of the animal (e.g., spatial coordinates, ordered by time) is termed a movement track or trajectory [17]. How well a trajectory reflects the actual movement path of an animal depends on the sampling regime as well as the recording systems (GPS, Argos, VHF, light-level geolocation), which influences the spatial accuracy and frequency of relocations.

In path-level movement data, consecutive relocations are either sorted by an ordering factor, for example as the result of direct tracking or following of an animal [18, 19] or by the time at which the relocations were recorded [16, 20]. Sampling frequency influences the resolution of the data and the level of inferential detail that can be obtained [5, 21, 22]. For example, shorter temporal intervals allow detailed insight into fine-scale behaviors, but are more sensitive to sampling errors (e.g., spatial inaccuracies of relocations). In contrast, movements sampled at longer temporal intervals can only be interpreted on a broader scale (e.g., encamped vs. dispersal movements). Additionally, recorded relocations can be spurious or lack spatial accuracy due to habitat induced sampling errors [23–26]. Importantly, trajectories also differ with regard to their regularity of the time intervals between successive steps. Irregular data commonly results from missing relocation fixes or varying sampling frequencies throughout a study period (e.g., [27]). Further, irregular intervals between relocation samples can stem from different behaviors of the study species. For example, relocation devices applied with marine animals can usually provide the measured position data only when the species is close to the surface [28–30].

### Basics of path segmentation

We use the term segmentation as a general paraphrase for determining changes in an animal's movement behavior based on the observed trajectory. The process of segmentation involves the partitioning of a trajectory, τ, into a number of *K* subtrajectories (τ_1_, τ_2_, …, τ_*K*_) called segments (Steps 1–3 in Fig. 1; see also [31, 32]). Path segmentation can be accomplished directly, by designating each observation to different states or clusters (e.g., [21, 33]). However, path segmentation commonly relies on detecting significant changes (so called change- or breaking-points) in the trajectory as cut-offs for separating the trajectory into distinct segments (e.g., [28]). For this, a variety of path characteristics can be derived from the trajectory, for example the step length or velocity. These path characteristics should accurately capture movement patterns and allow the detection of changes in these patterns. Given the importance of these path characteristics for successfully segmenting movement paths, we discuss them in more detail in the next section.

### Path characteristics

The various path characteristics used by current segmentation methods are summarized in Table 1. These characteristics have also been called movement metrics, movement parameters, path-signals or indices in the literature, and should convey relevant information about individual movement behaviors [31, 34, 35]. The majority of path characteristics are derived from consecutive relocations (stepwise), for example the speed of travel. However, some signals are calculated across multiple relocations, for example the straightness of a trajectory (Table 1).

**Table 1:** Currently applied path characteristics. Different signals or parameters can be calculated either based on consecutive relocations within a trajectory (“stepwise”) or for multiple relocations such as identified path-segments (“across multiple steps”)

Characteristic	Description	Type	Calculation	Reference
Displacement	Increment of the X and Y values between two consecutive relocations, change in absolute spatial position	primary	stepwise	[16, 34, 68]
Time lag	Duration / increment in time between consecutive relocations (usually determined by sampling regime)	primary	stepwise	[16, 34]
Turning angles / heading	Relative and absolute turning angles between consecutive relocations, change in direction	primary	stepwise	[16, 20, 37, 122]
Step length	Euclidean distance between two consecutive relocations	primary	stepwise	[16, 34]
Velocity / speed	Distance traveled in a given time interval between two relocations; less sensitive to missing data than step length	primary	stepwise	[16, 28, 34]
Persistence / turning velocity	Transformations of speed and turning angle: persistence velocity represents the tendency and degree of a movement to persist in a certain direction. Turning velocity shows the tendency of a movement to turn in a perpendicular/opposite direction	secondary	stepwise	[28, 35]
Net / mean squared displacement	Squared displacement between the first and current relocation of the trajectory; applied to characterize diffusion behavior or migration patterns	secondary	stepwise	[16, 20, 84]
First passage time	Time required for crossing a predefined endpoint based on a circle (radius) around a starting relocation. Sums the times of all forward and backwards relocations within the radius; index of area-restricted search behavior	secondary	stepwise	[31, 36, 123]
Residence time	Extension of the first passage time accounting for returns of the animal in a given area. Sums the times of all relocations (backwards and forwards) of a trajectory within a given vicinity around a relocation.	secondary	stepwise	[31]
Pseudo-Azimuth	Recalculates the basic azimuth value at the midpoint between two consecutive steps to range within 0 and 360. Can be used as indicators for movements with same or parallel directions.	primary	stepwise	[124]
Straightness index	Ratio of Euclidean distance between the beginning and end of a trajectory and the total path length (sum of all step lengths)	secondary	across multiple steps	[35, 123]
Sinuosity / Tortuosity	Adaptions of the straightness index analyzing the probabilistic distributions of the changes in the turning angles and the beeline distance between the start and end points of the trajectory; index of path orientation	secondary	across multiple steps	[38, 125]
Fractal dimension	Measure of path tortuosity; non-Euclidean dimension of the trajectory varying between one (completely straight) and two (tortuous, completely spanning two-dimensional space); different implementations exist	secondary	across multiple steps	[39, 126–128]
Multi-scale straightness index	Repeated calculation of the straightness index of a trajectory over a range of different temporal scales	secondary	across multiple steps	[76]
Area interest index	Repeated calculation of the straightness index for a limited size of a sliding window along the trajectory. With each repetition, the number of relocations within the trajectory is reduced	secondary	across multiple steps	[76, 77]

Dodge et al. [34] distinguished primitive path parameters from primary and secondary derived parameters. The information on the absolute spatial position (e.g., xy-coordinates) and the temporal dimension (time stamp) provide the primitive signals from which other parameters can be derived. For example, displacement and step length (see Table 1) are primary derivatives of the position parameter, whereas time lag (duration) is derived from the temporal primitive.

Path-signals exclusively based on spatial criteria are particularly sensitive to sampling intervals and errors [16, 21]. However, other signals such as the persistence or turning velocity avoid possible biases caused by varying sampling intervals by relating speed to the observed turning angles. Furthermore, signals such as the first passage [36] and residence time [31] constitute summary properties accounting for the temporal scales within the movement paths and can be seen as secondary derivatives of the distance and duration signals.

Table 1 also lists characteristics which are calculated over multiple relocations and can be applied to describe the signals of single segments, certain sub-samples of trajectories, or entire trajectories. Such summary signals like the straightness index [37], sinuosity [38] and the fractal dimension [39] provide information on the spatial complexity of a given path segment and can be used to cluster segments into groups that are similar with respect to movement complexity (Step 4 in Fig. 1). Sinuosity constitutes another example of a secondary derivative of the step length signal [34].

Overall, a large number of different measures can be used to describe path characteristics and a chosen parameter should ideally convey relevant information about the underlying movement behavior [31]. This requires a good understanding of the species and a precise definition of research questions, and should also involve extensive exploratory analyses to understand the structure of obtained relocation data and to test the feasibility of different segmentation approaches (Step 2 in Fig. 1; see also below and [35]).

### Finding and interpreting segments

Regardless of how and which path characteristics are quantified, significant changes within these signals are then used to determine the *K*-1 break-points (τ*_1_, …, τ*_K-1_) which can be used to divide the trajectory into *K* segments (Step 3 in Fig. 1). Although preliminary visual analyses can provide useful indications about a meaningful value for *K*, an objective, data driven way is desirable. Therefore, path segmentation often involves quantitative approaches for detecting an unknown number of segments within a given trajectory, and many of these approaches have originated in non-ecological disciplines (e.g., [40]). This is an important point, as many segmentation methods only provide information on significant change-points along the trajectory, without any further ecological context. Thus, it is often not trivial or even possible to directly associate the individual segments to specific activities and behaviors [41]. To facilitate the ecological and ethological interpretation of the defined segments, some methods require subsequent analyses to classify the determined segments based on different descriptive parameters or summary statistics (Step 4 in Fig. 1). For example, either the mean values of stepwise characteristics or multi-step summary parameters, such as the straightness index (see Table 1), of the segments can be further analyzed in an additional classification analysis (e.g., [41]). This generates clusters of segments that are similar with respect to relevant path parameters (e.g. calculated across multiple steps, Table 1), which can help to identify underlying movement patterns and associated behaviors. For example, short, meandering movement segments during within-patch foraging vs. long, straight segments during inter-patch movements [42, 43]. Other methods determine the state (also called class or cluster) of each individual relocation directly and no further classification is necessary [21, 33].

In sum, path segmentation involves at least three and sometimes four major steps (Fig. 1). In the following, we focus on the third step, in which signals derived from trajectories are used to objectively define movement segments.

## Overview of path segmentation methods

### Types of methodological approaches

Methods for path segmentation can be distinguished or classified using many different criteria, for example based on their underlying statistical framework (e.g., maximum-likelihood versus Bayesian; parametric or non-parametric, inference-based etc.). Alternatively, Gurarie et al. [35] recently classified broad types of movement analysis tools based on the analytical traditions they stem from. Since our overview is specifically intended for beginners wanting to apply path segmentation, we do not categorize methods based on their statistical properties or analytical traditions, but instead focus on the practical utility of the analyses, e.g., the research questions that can most readily be answered with a certain approach. Hence, we structure our overview based on three broad types of questions that are commonly addressed using path segmentation.

**Fig. 2:**
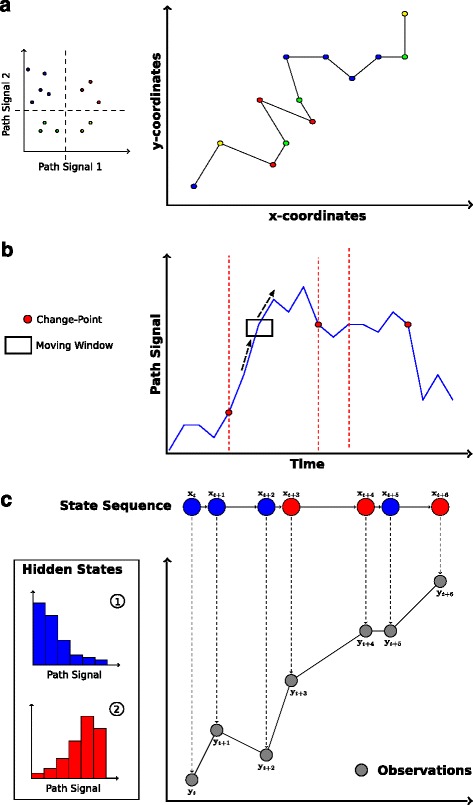
The main study aims of path segmentation and types of methods to address them. **a** Pattern description: Topology-based analyses rely directly on signals calculated from the movement trajectory (e.g. step length and bearing). They combine movement steps into groups based on similarity in the considered path-signals, for example by applying clustering algorithms. **b** Change-point detection: Time-series analyses assess a path-signal (y-axis) along its time-axis. For example, a moving window (*rectangle*) can be used to search for points along the time-series where local parameters (e.g. the mean) of the path-signal are significantly different from the global averages of these parameters. Significant change-points are assumed to indicate switches in underlying movement modes or behavioral states, and are used to separate the trajectory into segments (*dashed lines*). **c** Process identification: The majority of the presented state-space models link two stochastic models describing the state process and its observation. For example, the state process could consist of two discrete behavioral states (*red* and *blue*). The process model describes how the hidden state (x) emerges based on a Markov process. Therefore, it accounts for the conditional probability of a future state depending on the one of the current relocation. The observation model links the actual observed data (y) at given points in time to the hidden state. As a result, the most probable state of each observation, the switching probabilities between the states, as well as the distributions of the measured path-signals within each state are provided.

First, movement patterns within the trajectory can be quantified to test whether different movement components are identifiable within the data. For example, such ‘*movement pattern description*’ is used to distinguish active from resting phases (e.g., [44]), or encamped foraging from traveling movements (e.g., [45]). Second, path segmentation can also be used to locate significant changes in movement behavior and determine the timing of these changes. For example, such ‘*change-point detection’* has been used to quantify behavioral responses to seasonal environmental changes (e.g., [46]), or to identify the timing of migration events (e.g., [47]). Finally, path segmentation can be used to take a detailed look at the processes underlying observed movement patterns. Such ‘*process identification*’ can be used to examine the factors influencing diel variation in movement rates among individuals (e.g., [48]), or to quantify how sex and reproductive status influence the duration of, and transition among, different behavioral modes [49]. These three broad types of research questions can be matched to three basic categories of analytical approaches for path segmentation (Fig. 2).

### Topology-based approaches to describe movement patterns

If the study aim is to quantitatively describe movement patterns, one can use methods that focus on the description of geometric properties of the trajectory itself, or on one or several signals calculated from the trajectory. Based on this path topology, movement steps are then assorted into groups that are relatively similar with respect to these signals (Fig. 2a). The exact way this is accomplished depends on the method, but can be achieved either by *a)* simply grouping individual movement steps based on similarity in topology-based signals, regardless of whether these steps are consecutive (e.g. thresholding or clustering; [21, 45]; or *b)* identifying changes observed among the signals between successive relocations to detect so-called change-points (e.g., spatio-temporal criteria segmentation; [32]). These change-points are assumed to correspond to changes in underlying movement behavior, therefore separating the trajectory into segments consisting of multiple consecutive steps based on pronounced changes in observed movement characteristics. These topology-based methods are mostly non-parametric and rather descriptive. Their application is usually based on predefined hypotheses on how movement behaviors might differ among habitats, seasons, times of day, sexes, social status, etc..

### Time-series analyses to detect significant change-points

If the goal of a study is to detect points in time when a significant change in the movement behavior occurs, path segmentation methods based on time-series analyses can be used. Such time-series analyses are widely used in ecology and related disciplines (see [50]). In the context of path segmentation, these analyses treat signals calculated from consecutive movement steps as time-ordered observations. Essentially, the majority of these approaches try to find significant change-points along the time axis of the signal-sequence derived from the movement trajectory (Fig. 2b). In contrast to the topology-based approaches that analyze the changes between temporally ordered relocations, most of the time-series methods treat movement patterns as a function of time and can directly account for the temporal correlations of the sequential signal data. The time-series approaches sometimes depend on certain information like the maximum number of change-points or the minimum length of the detected segments. However, they could also potentially be used to “blindly” search for all possible change-points of a given path-signal sequence.

### State-space models to identify underlying processes

Finally, to increase our understanding of the behavioral processes underlying complex movement patterns, methods derived from the state-space modeling framework are most suitable. These state-space models represent a special type of time-series analysis [51] and intend to identify latent or hidden behavioral states based on the observed movement data. The aim is to derive deeper insight into the underlying processes by formulating a movement model that explains observed movement patterns. Within these frameworks, the future state of a system is modeled to depend on its current state through a probabilistic model (see Fig. 2c). Therefore, the models typically assume a so-called Markov process structure, meaning that a hidden future state depends on the state of the current step [52]. Essentially, state-space models couple two stochastic time-series models, one based on an unobservable state process, and another based on a known observation process [52, 53]. When applied to movement data, state-space models assume that animals have several ‘hidden behavioral states’ with certain characteristics (e.g., path-signals) that can be modeled using stochastic processes (e.g., correlated random walks; [54]). A basic result of a state-space model are the estimated transition probabilities between the considered states. Another outcome is the probability of a given relocation belonging to one of the hidden behavioral states. These probabilities are then used to assign steps to their most probable behavioral state (Fig. 2c) and to segment the trajectory according to state memberships. Additionally, the transition probabilities can also be linked to different environmental factors to test various hypotheses on behavioral and ecological dependencies of the observed movement patterns [54–56]. For example, the transition probabilities can be used to test whether switching between states depends on certain habitat characteristics (see simulation study below).

## Choosing among methods for path segmentation

Multiple methods for path segmentation exist within each of the three types of analytical approaches described above. Thus, multiple methods exist to answer each of the broad categories of research questions (study aims). Table 2 provides an overview of the available path segmentation methods and lists basic properties, and important background papers for each method. More detailed descriptions and further information on each path segmentation method, including implementations in the program *R* [57], can be found in Additional file 1: S1.

**Table 2:** Characteristics of the methodological approaches for the three different categories of research questions. Different methods for answering the three type of broad research questions (study aims) are listed together with the analytical category they stem from, a short description of each method as well as the considered categories of input path-signals and important references

Study aim	Method	Analytical category	Description	Input signal	References
Movement pattern description	Thresholding	Topology-based	Applies thresholding schemes (cut-off values) to separate relocations into different groups based on single or multiple path parameters (e.g., short- vs. Long-range movements)	Primary and secondary signals	[45, 80, 84, 127]
	Supervised Classification	Topology-based	Relocations (steps) of a trajectory are assigned to certain classes of movement behavior based on a classification scheme fitted with a training dataset	Primary and secondary signals, additional information like activity data	[129–131]
	Clustering	Topology-based	Unsupervised classification for identifying distinctive groups within a multivariate set of path-signals	Primary and secondary signals, additional information like activity data	[21, 132]
	Bayesian Partitioning of Markov Models (BPMM)	Topology- and time- series based	Classification algorithm for determining the number and sequence of homogenous classes within a sequential path-signal (time series)	Primary and secondary signals	[35, 91, 92]
Change-point detection	Line Simplification	Topology- or time-series based	Tests whether reducing the number of vertices in a trajecotry significantly impacts path topology to determine change points (can also be applied with graphs of sequential path-signals)	Primitive signals (spatial position)	[12, 133]
	Change Point Test	Topology-based	Detects significant changes in the observed movement direction (orientation) between the starting point and an attraction point of a trajectory	Primitive signals (spatial position)	[86, 134]
	Spatio-Temporal Criteria Segmentation	Topology-based	Special type of thresholding seeking optimal segmentation of a trajectory based on monotone criteria: relocations are included in a segment as long as they fullfill certain predefined requirements	Primitive, primary and secondary signals	[32, 87]
	Piecewise Regression	Time-series analysis	Splits time-series model into representative segments based on a signficant change-point (fits a polynomial model for each segment)	Primary and secondary signals	[86, 87]
	Penalized Contrast Method (PCM)	Time-series analysis	Non-parametric segmentation of a path-signal: the unknown number of segments is estimated by minimizing a penalized contrast function	Mostly secondary signals	[31, 40, 135]
	Behavioral Change Point Analysis (BCPA)	Time-series analysis	Likelihood-based method for detecting significant change points; applies moving window over continuous autocorrelated time series of a path-signal	Mostly secondary signals	[28, 35]
	Pruned Exact Linear Time (PELT) Algorithm	Time-series analysis	Search method for detecting optimal number and locations of change points minimizing different cost and penalty functions	primary and secondary signals	[42, 136, 137]
	Behavioral Movement Segmentation (BMS)	Time-series analysis	Combined search algorithm which optimizes segmentation based on parsimony and subsequent clustering for assigning segments to similar behaviors	primary and secondary signals, additional information like activity data	[43]
Process identification	Hidden-Markov Models (HMM)	State-space models	Estimate the sequence and composition of a predifined number of discrete states (e.g., movement behaviors) as well as the switching-probabilities between these states	Primary signals, additional information like activity data	[33, 49, 53–55]
	State-Space Models with Location Filtering	State-space models	More complex models which can model hidden movement states and also correct for errors in the observation process (e.g., GPS errors)	Primitive (spatial position) and primary signals, additional information like activity data	[51, 52, 65, 88, 90, 138]
	Hierarchical State-Space Models	State-space models	Hierarchical models accounting for variability of number and composition of movement states between individuals (further making inferences at population level)	Primary signals	[48, 52, 89]
	Bayesian Partitioning of Markov Models (BPMM)	Topology- and time- series based	Can also be used as partitioning algorithm determining the number and sequence of homogenous models (“states”) within a sequential path-signal	primary and secondary signals	[35, 91, 92]

**Fig. 3:**
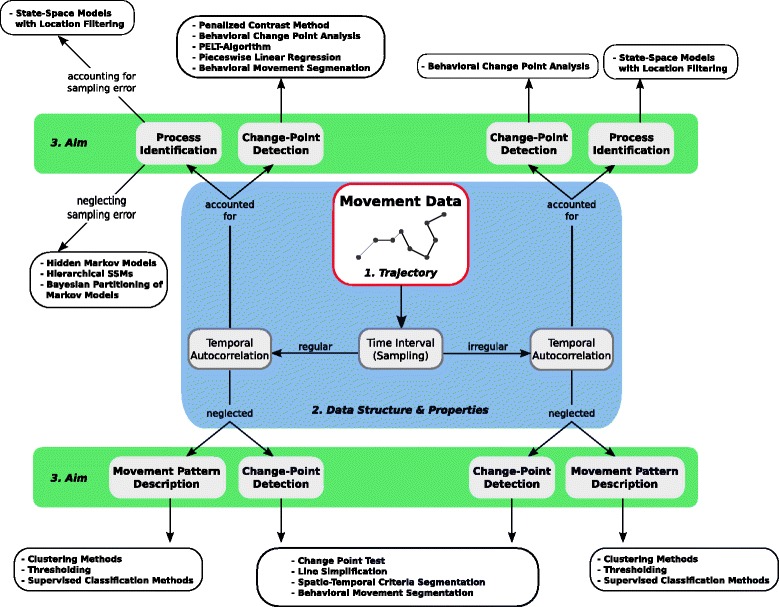
Decision guidelines for choosing appropriate segmentation methods. The process should begin with preliminary analyses of the trajectory data and derived path-parameters (1). Choosing among methods is then first directed by the data structure and sampling regime (2). Capability of the methods to account for temporal autocorrelation further determines the decision process. In the end, study aims and objectives guide the final decision on a given segmentation method (3)

Available path segmentation methods vary substantially with regard to their demands on data structure and underlying theory. This raises the question of how scientists can identify the most appropriate segmentation method(s) for their specific research goals. In the following, we provide some general guidelines for method selection. Additionally, the guidelines are visually summarized in Fig. 3.

### Preliminary data analyses

Because the structure and composition of movement data dictate the applicability of certain methods (Fig. 3; blue panel), the first step in any segmentation study should be a preliminary analysis of the available location data. Various analyses can be carried out to gain a better understanding of data properties, but a preliminary analysis for path segmentation should contain at least the following four steps.

1. Sampling regime

Movement data usually varies substantially with regard to the sampling regime, spatial accuracy and temporal resolution. Therefore, preliminary analyses should include checking for regularity of time-intervals between relocations as well as testing for temporal autocorrelation of the path-parameter data [16, 58]. Depending on the results of these analyses, several segmentation methods may no longer be suitable (Fig. 3).

2) Data regularity

Irregular data can be the product of missing relocation fixes and varying sampling regimes which can be a challenge, as some of the statistics used to analyze movement paths assume regular intervals within the trajectory and are valid only under those circumstances [28]. Different processing tools can be applied to relocation data in order to fulfill the assumptions of regularity. For example, trajectories can be re-discretized [16, 38], which means that relocations can be removed until the remaining data fulfills the requirement of temporal regularity (“thinning”). Alternatively, missing relocations can be replaced by applying techniques such as spatial interpolation [12, 59] or dead-reckoning [60–62]. Furthermore, only subsets limited to continuous and regularly sampled relocations of the original trajectory can be selected for further analyses [16, 38]. Approaches modeling movement in continuous time are also capable of dealing with irregular data structures (e.g., [53, 63]).

Additionally, habitat induced sampling errors and spatial inaccuracies can occur and need to be addressed throughout the preliminary analyses [23–25]. This includes checking the data for extreme outliers or estimating the error of the applied tracking technology (e.g., provided by ARGOS systems; [64]). Some types of state-space models include location filtering where such information can be implemented as a prior in order to estimate the true positions of erroneous relocation data (e.g., Kalman Filtering [65–67]).

3) Data visualization and signal distributions

Visual inspection of the movement trajectory can already indicate the existence of different behavioral modes [68–70]. Also, in order to choose appropriate path-signals conveying information on potential changes within the movement behavior, investigations of their variability and distributions (e.g., histograms) should be considered. For example, multi-modality within the path-signal distributions can also indicate the potential existence of different behavioral modes (see applied examples). Further, depending on the intended segmentation method, knowledge on the parameter distributions is also needed in order to fit movement models within the various types of state-space models [54, 71]. As a substantial part of the methods stem from the time-series framework, time-ordered plotting of the path-signals can indicate the existence of changes in the sequence over time (see applied examples below). Visual inspection of the variation of the signals over time can provide insight on the ranging and movement behavior. For example, Bunnefeld et al. [72] and Killeen et al. [15] inspected time-ordered values of net-squared displacement (Table 1) for single or multiple modality in order to detect potential migratory individuals.

Further, the visual inspection of movement trajectories can help to identify unusual relocations and movements [69, 70]. Thus, visual inspection of the trajectory is important for error checking and can help to refine biological hypotheses to be tested with a given data set.

4) Scales of movement and data transformation

Detectability and observability of changes in movement behavior can also change with temporal and spatial scale [18, 73]. There are multiple options of indexes and transformations providing information on the varying spatial and temporal scales of the path characteristics (e.g., trigonometric circle space; [12]). Further, sub-sampling, re-discretization or moving-windows can be applied to alter the temporal grain (e.g. daily, nocturnal, weekly or monthly relocations) in order to summarize the means or variances of path-parameters [22, 29, 74, 75]. Also, multi-step signals (see Table 1) such as the simple straightness index [37] and its different extensions [76, 77] can be applied to investigate the variation of path straightness within a trajectory over time and multiple temporal resolutions. Path-parameters such as the first passage or residence time (Table 1) can be calculated at varying spatial and temporal scales and allow further insight in underlying spatial and temporal scales of individual movement behavior [31, 78, 79]. Finally, different transformations of the path parameters can be applied to determine dominant and constant periodic frequency patterns in the movement data. For example, Fourier and wavelet transformations provide valuable insight in periodic structures of movement, such as circadian, seasonal or diurnal rhythms [80–83].

### Study aims

After the preliminary analysis of the data structure and relevant path characteristics, choosing appropriate segmentation methods is mostly influenced by the aims of the study (Fig. 3; green panels). Thus, depending on the study aims and data structure, different methods can be applied.

1. Movement pattern description

The majority of appropriate methods for quantitatively describing movement patterns are based on the path-topology approaches such as simple threshold or multivariate classification algorithms (detailed information for each method in Additional file 1: S1). These approaches are least demanding with regard to data properties like regularity and do not require any data transformations as they make minimal assumptions about underlying data structures, movement models, or behavioral states. However, they can be valuable exploratory tools for determining the potential number of different behavioral states within the observed movement data (e.g., [21, 45, 84]). Furthermore, the methods can be applied for testing certain hypotheses on how particular path-signals change with different behaviors or at certain time-periods. Therefore, for some study aims it might be sufficient to split movements into two or more different behavioral states (e.g., long- vs. short-range movements) based on a threshold within a selected path-signal (e.g., step length; [85]). Similarly, the time when the relocations were recorded could be used to distinguish different types of behavior (e.g., daytime vs. nocturnal movements).

In sum, methods for pattern description can be applied to gain insight on potential behavioral states and even for detecting potential drivers of the observed patterns (e.g., nocturnal movement behaviors with longer step length). However, the considered path-signals have to be chosen carefully and according to expected changes in movement behaviors and underlying behaviors [21, 35]. Furthermore, due to their relative simplicity, topology-based methods offer little explanatory power and are usually not suitable for analyzing complex movement patterns [35].

2) Change-point detection

The second example of a general study aim is the determination of important (significant) change-points in the movement behavior or trajectory of an animal. The presented approaches either focus on the path-topology or on a time-series of a path-signal. In both cases, the sequential relationship between consecutive relocations is accounted for.

The relevant topology-based methods either focus on the changes within the absolute spatial position (e.g., the change point test [86]; Table 2) or different path-signals and their shape along the trajectory (e.g., using Spatio-Temporal Criteria Segmentation [87]; Fig. 3). However, the change-points resulting from the topology-based methods usually do not provide any information on the significance of the observed changes within the data composition. If identifying significant change-points is the aim, for example, to detect the onset of migratory events, then methods from the time-series category are the better choice, as they specifically estimate the significance of changes within a time-ordered data sequence (Fig. 2b). The majority of time-series approaches are capable of accounting for temporal autocorrelation within the data sequence which can be an important advantage, because non-independence of relocations is a challenge for many standard statistics [28]. As can be seen in our example, the autocorrelation structure of the data can also contain valuable information about the underlying behavioral states [13]. As a caveat, most time-series methods show higher demands on data properties, especially regularity of the time intervals between relocations (an exception is the behavioral change-point analysis; BCPA). Furthermore, many of the appropriate time-series methods listed in Table 2 depend on one or multiple parameters which need to be defined prior to the analyses such as the size of a moving window (e.g., for the behavioral change point analysis; [28]) or the minimum number of relocations within a determined segment (e.g., for the penalized contrast method; [40]). Therefore, several assumptions, about the number of potential changes or the length of a behavioral state, need to be made before setting these parameters, which increases the susceptibility to errors and bias and limits reproducibility.

In contrast to that, topology-based methods for change-point detection are less dependent on such parameter settings and mostly focus on changes within the spatial composition of the trajectory. However, the scale at which these methods can detect changes in movement behavior is highly dependent on the temporal resolution of the data. Relocations recorded at higher frequencies can provide more detailed information on fine-scale behaviors. Low frequencies usually limit the scale at which the topology-based algorithms can determine changes in the underlying behavior [17, 86].

Time-series approaches are usually less sensitive to the temporal sampling frequency of the data for detecting change-points when appropriate input signals conveying meaningful information are used (e.g., persistence velocity; [28]). However, time-series based methods need to be chosen carefully as their assumptions on data distributions (e.g., Gaussian vs. non-Gaussian time-series) and applied statistics can differ (see Additional file 1: S1 for more details).

3) Underlying process identification

To identify processes underlying complex movement behaviors, various types of state-space models (SSM) are suitable choices. SSMs intend to identify latent states or hidden models based on the observed movement data. In this context, hidden states represent different behavioral modes, assuming that they can be described with different parametric distributions of the path characteristics. The majority of SSMs can be interpreted as a multi-state random walk and are usually based on assumptions about the density functions of the step length and turning angle distributions [35, 54]. Hierarchical approaches can be used to estimate different numbers and compositions of behavioral states for each of the studied individuals and further draw model inferences at the population level [52, 54, 88, 89]. Another advantage of these models is that some can account explicitly for issues of animal movement data, such as irregularities caused by missing relocations and measurement errors (e.g., location filtering [51, 52]). In particular, SSMs fitted with Bayesian estimation techniques allow the integration of prior knowledge on sampling errors [25, 51, 88]. For example, information on the accuracy and quality of the acquired relocation data as provided by the ARGOS system can be implemented in the observational model of such a SSM framework [67, 88, 89]. Importantly, state-space models can integrate the influence of habitat features and other environmental information, such as sea depth or temperature obtained from electronic tagging data, on behavioral changes [53, 55, 90]. Therefore, they provide a valuable framework for estimating and comparing the responses of state compositions and their transition probabilities to different covariates [49, 54, 56]. Furthermore, due to their mechanistic basis, many of the SSM methods provide information on the differences in the estimated parameter distributions of the considered movement models. Thus, state-space models can also be used to simulate or predict movement patterns under varying environmental settings [51]. The biggest challenge of using state-space models is the necessity to estimate the various model parameters, which can require mathematically and computationally complex procedures [48, 53]⁠. In summary, state-space models offer much flexibility towards a mechanistic understanding of animal movements, because the process models make it possible to fit specific underlying movement patterns (e.g., different correlated-random walks) to the observed movements [51, 88]⁠.

However, the number of potential states considered within the models usually needs to be determined prior the application [53]. Also, the general composition of the considered movement models within the states has to be predefined. This limits SSM mostly to variations of discrete correlated random walks [54].

Another option for identifying “hidden states” with different compositions of movement parameters is the Bayesian partitioning of Markov models (BPMM) [35, 91]. Technically, this approach is not a state-space model but it represents a simple solution for detecting different models within the observed movement data. The method estimates the distributions of a path-signal for a given number of potential states and assigns each relocation to one of them [91, 92]. However, BPMM does not provide any information on the potential processes, the transition probabilities between the detected states, or the potential influence of covariates.

## Illustration using simulated data

**Fig. 4:**
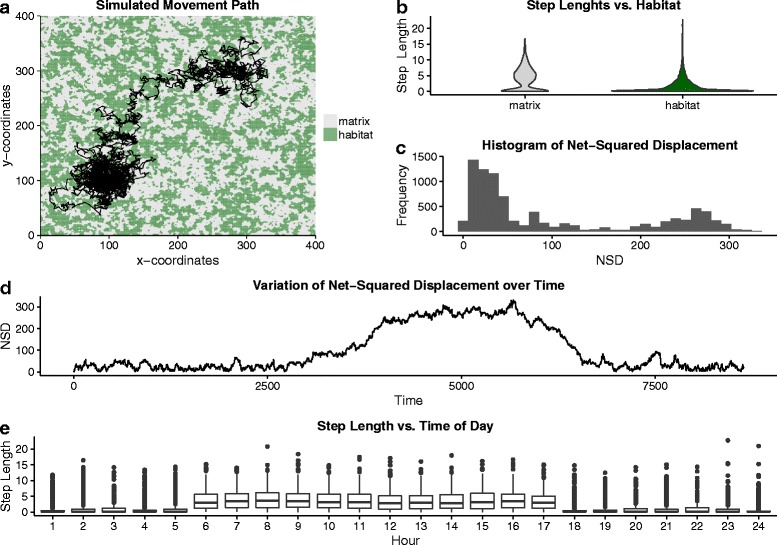
Simulated trajectory and results of preliminary analyses. **a** overview of the simulated movement path and habitat configuration. **b** distributions of observed step lengths within and outside the habitat (*matrix*) of the tracked animal. Results of preliminary analyses for the net-squared displacement signal including the distribution (**c**) and the time-series across the entire tracking period (**d**). Distributions of observed step lengths at different hours of the day (**e**)

To illustrate the three types of research questions and related analytical approaches, we next apply one method of each type of analytical approaches to a single data set. For this, we used a simple individual-based simulation model to generate the annual movement track of an animal in R [57]. Details on the simulations and all relevant parameters can be found in Additional file 2: S2. In essence, we simulated an animal that is more active during the day than during the night, moved faster in its habitat than in the matrix (unfavorable habitat) and migrated between two centers of activity (e.g., summering and wintering range). We simulated a movement track for 12 months with relocations taken every hour in a landscape consisting of 400 * 400 cells (Fig. 4a).

For this data set, we were interested in three different research questions. First, we evaluated the hypothesis that the movement intensity of the animal somehow differed between its habitat and the (potentially hostile) matrix, *sensu stricto* non-habitat. To address this question, we chose a topology-based method using a threshold to distinguish short- from long-range movements and compared the proportions of these two stages within the habitat and matrix. Second, we wanted to assess whether the animal showed a seasonal migration pattern and, if so, to detect the times when migration movements occurred throughout the year. For this, we applied a time-series analysis to segment the movement data based on changes in an observed path-signal. Finally, we assessed whether two different behavioral states could be distinguished and whether the switching probability between those two states could be linked to time of day and habitat. To answer this research question, we used a state-space model approach with two discrete states differing with regard to their distributions of certain path parameters. Before addressing these research questions, we performed different preliminary analyses to gain insight about data properties and guide the decision process on meaningful path-signals and an appropriate segmentation method for each question (Fig. 3).

### Results: preliminary analyses

As pointed out above, preliminary analyses are a fundamental part of path-level analyses and should be performed thoroughly prior to the application of any segmentation approach. As our simulated data set consisted of relocation data sampled at an hourly interval, we did not test for regularity of the sampling regime. However, such tests can be performed by inspecting the distribution of the time-lags between the sampled relocations (e.g., using histograms). More analyses for checking the regularity of a trajectory or testing the independence of missing data points are implemented in the *adehabitatLT* package [92]. In the next step, one should test for potential correlation structures within the observed movement data. We applied different tests based on Dray et al. [58] and detected significant correlations between consecutive measures of the step length and also turning angles up to a time lag of five relocations. Therefore, following our guidelines (Fig. 3), we chose among methods accounting for such temporal autocorrelations.

Meaningful path parameters conveying relevant information about potential changes in movement behavior are essential for a sound path-segmentation analysis. Thus, comparisons of different signals (e.g., primary and secondary derivatives, Table 1) with regard to their distributions and variation over time should be performed in the preliminary analysis. We applied several exploratory analyses for the step length (due to the hourly sampling regime this is also the speed signal), turning angles and net-squared displacement (NSD) signals (more details in Additional file 2: S2). For example, Fig. 4 shows the distributions of NSD and step length as well as their variation over time. The NSD signal provides meaningful information on the ranging behavior of an animal as it represents the distance to the point where the tracking period started. Inspection of this signal over the entire sampling period revealed that there was a steep increase in the values of this parameter followed by a plateau and decrease until the values were in the same range as at the beginning (Fig. 4d). Further, we observed a trend for a bimodal distribution of NSD (Fig. 4d). As described above, behavioral changes might be detectable at different temporal scales. Plotting the distribution of step lengths against the time of the day they were recorded revealed that the animal was potentially more active during the day as during the night (Fig. 4e). Finally, we used all three path signals, step length (in our case equivalent with speed), turning angles and NSD for the different segmentation approaches.

### Results: habitat-specific movement patters

**Fig. 5:**
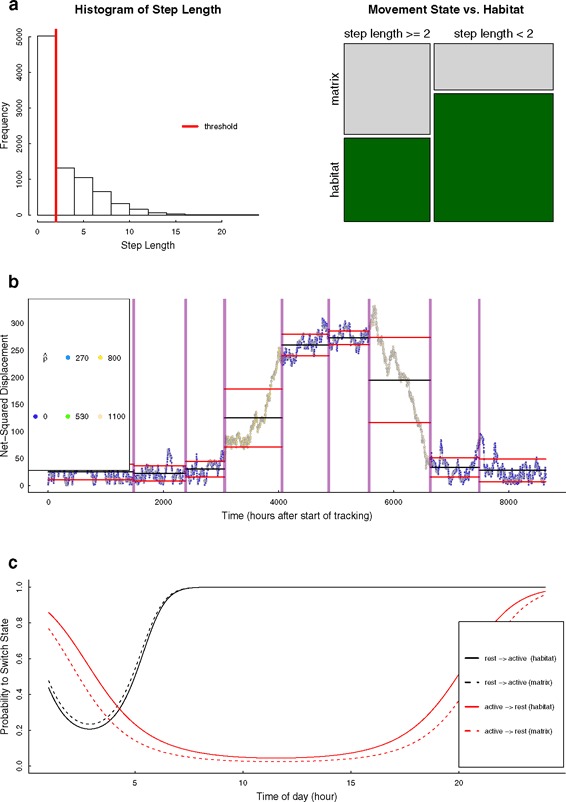
Results of three different segmentation methods using the simulated movement data. **a** The left panel shows the distribution of the observed step lengths as well as the applied cut-off value (threshold = 2 units). The proportions of the resulting behavioral states (short- and long-range movements) within and outside of the habitat are shown in the right panel. **b** Results from the behavioral change point analyses applied with the net-squared displacement signal. The observed time-series was segmented at significant change-points (*vertical lines*) to distinguish movements within the main ranges of the animal and two migratory periods. The color of the estimated parameter ρ^ indicates the level of temporal autocorrelation. **c** Change in switching probabilities between the two states (resting vs. active) dependent on the different hours of the day. Switching probabilities also differed with regard to whether the animal was in its habitat or not. Black lines indicate the switches from the resting state to the active state. Red lines are showing the switching probabilities from active to resting state

We applied a thresholding method to distinguish two different movement patterns within the simulated dataset. A simple cut-off value was used to split relocations into short-range (e.g., encamped) and long-range (e.g., roaming or dispersing) movements. Relocations with an observed step length shorter than two units were considered short-range movements whereas those with a longer step length were classified as long-range movements. As can be seen in Fig. 5a, the proportion of the two movement behaviors varied between habitat and non-habitat. For example, the majority of short-range movements (about 73.3 %) occurred within the habitat of the animal. More than half of the movements (about 58.5 %) outside the habitat stemmed from the long-range behavioral state. Further, a chi-square test indicated a significant (non-random) distribution of the two stages between habitat and non-habitat (*p* < 0.001). Clearly, results highly depend on the chosen threshold value. Therefore, cut-off values need to be chosen carefully and based on well-reasoned inferences, especially when they are applied with real movement data (see examples in [45, 85]).

### Results: timing of migration

In our applied example, we chose the behavioral change-point analysis (BCPA [28]; see Table 2) to demonstrate how significant changes can be detected within a time-series of a path-signal in order to find segments of potential migratory behavior. We chose the sequence of the net-squared displacement parameter (NSD, Table 1) as the model input. As can be seen in Fig. 5b) the BCPA determined multiple segments with comparably low net-squared displacement prior to the simulated migration event (from 0 to 3000 h after the start of tracking). That period is followed by a segment with increasing displacement and also higher autocorrelation which can be interpreted as potentially migratory behavior. The plateau within the NSD time-series (around 4000 to 5500 h after start of tracking) marks the arrival of the simulated animal track in its second range (e.g., summering grounds). The second migratory event is once again detected by a segment with decreasing NSD but also high autocorrelation values. Finally, the last two segments have low values of NSD comparable to the beginning indicating that the animal has returned to the first range where the tracking was started (e.g., wintering grounds). In summary, the time-series based analysis was successful at determining multiple segments, including a distinction of within-range movements from migratory movements, as well as an identification of the starting time of migration.

### Results: underlying processes

In the third example, we addressed the question whether the switches between different movement states could be linked to two covariates, the time of the day and whether the animal was within or outside its habitat. We applied a hidden Markov model (HMM; Table 2) with two discrete behavioral states which differed with regard to their means of the step length and turning angle parameter distributions (more details are presented in Additional file 2: S2). The model was fitted using the *moveHMM* package [93]. The first state consisted of relocations with very low step length values (mean of 0.11 units) and mostly negative turning angles. Therefore, this state was considered to represent resting or sedentary movement behavior. In contrast, the second state comprised of relocations with longer step lengths (mean of 3.4 units) and positive turning angles potentially representing active movement phases. The probability for the animal to switch from the resting to the active state was lower during the beginning of the day and increased with daytime (Fig. 5c). The switching-probability from active to resting decreased during daytime and was higher during the night. Further, the probability to switch from resting to active was slightly higher when the animal was outside its habitat. Complementary to that, the animal was less probable to switch from active to resting when it was in non-habitat (Fig. 5c). Overall, the model output represents the simulated movement behavior which consisted of higher movement activity during the daytime and faster movements outside the habitat. This underlines the high potential of different state-space model approaches for gaining a better understanding of the processes and mechanisms potentially driving the observed movement patterns [35, 51].

## Discussion

The aim of movement ecology is to gain a deeper understanding of the mechanisms and ecological processes shaping organismal movement patterns and their consequences for ecology and evolution [4, 5]. The methods presented here can be applied to define behavioral states from the observed movement paths and link these behavioral states to different environmental covariates to gain an enhanced understanding of the biological processes influencing the movement behavior of animals [14, 15]. However, there is no single method that can be universally applied to any kind of study scenario. As illustrated above, path segmentation methods vary substantially with regard to their demands on data structure and underlying theory. Given this analytical variability, there are certainly several possibilities on how to group and categorize the different methods for path segmentation (e.g., [35]). Here, we chose to contrast different analytical approaches with regard to their applicability for answering certain research questions, rather than their underlying statistical frameworks. Nevertheless, we encourage researchers interested in applying path segmentation methods to read about the statistical details of the different methods (Additional file 1: S1) and consult the original method papers to fully understand the statistical properties of the method(s) they intend to apply.

We used a simulated dataset to demonstrate how our proposed decision process (Fig. 3) can be performed to answer different research questions using methods from the three analytical categories of topology-based, time-series and state-space analyses. Certainly, each of these categories have advantages and disadvantages one has to account for when choosing among them.

The majority of methods focusing on path-topology (Table 2) are purely descriptive and usually just draw new observations based on the tracked movement pattern [33, 35]. However, for certain analyses this might already be sufficient to answer the defined research questions. For example, we showed how a relatively simple thresholding approach can be used to distinguish between two extrema of a potential movement behavior (short- vs long-range movements) based on a path characteristic and linked them to different habitat configurations. Thus, topology-based approaches are useful when specific hypotheses regarding movement patterns can be formulated *a priori* [21]. Also, topology-based methods are least demanding in terms of data composition and regularity, as they make no specific assumptions about data properties or the distribution of the considered path characteristics. Furthermore, they are analytically the most straightforward and can serve as exploratory tools e.g., for determining the number of potential movement states that could be further analyzed in a more inference- or process-based approach such as a SSM [33]. However, these methods should not generally be applied as end-point analyses since they are mostly ignoring other valuable information like the serial autocorrelation of path parameters.

Time-series based approaches are usually more demanding with regard to data composition but provide deeper insight to significant changes in movement behaviors and account for important correlation structures present in movement data [28]. Such methods can easily be used for finding single or multiple change-points in a trajectory to determine the moment of important changes in movement behavior.

State-space models are arguably the most powerful way for analyzing animal movement data, providing a “bottom-up” (holistic) approach where behavioral states and switching probabilities between them are modeled within the same process [51, 52, 55]. However, the estimated state configurations are also based on certain model assumptions about the movement properties (e.g., variants of correlated random walks) and the observed pattern in the considered data [53]. Therefore, SSMs do not ultimately convey a biological meaningful differentiation between different (“true”) movement behaviors [53, 55]. Furthermore, many of the presented SSMs are quite complex and hence perhaps the most challenging to apply to empirical data. In order to foster the application of state-space models in movement ecology, we encourage biologists to cooperate with statisticians and modelers when designing studies and analyzing data. Such interdisciplinary research teams should refer to the growing number of *R* packages for fitting state-space models (e.g., [93, 94]; see Additional file 1: S1), and to the increasing number of papers providing practical advice for using these models (e.g. [51, 52, 95]).

Finally, the majority of the presented methods of the time-series and state-space analyses are based on discrete-time models and therefore require regular sampling regimes (Fig. 3; [96]). Such data regularity is not always possible to obtain, even though various procedures reaching regular sampling are available (see above). However, there are multiple approaches using diffusion processes which model movements in continuous time and are capable of dealing with irregular data compositions [53, 97]. For example, highly infrequently sampled movement data can be analyzed using a spatial HMM with a discrete space structure [52, 98]. Furthermore, methods implementing continuous time processes and estimating switches between different behavioral states were presented by [63, 99–102].

As highlighted by Gurarie et al. [35], preliminary data analysis is a very important part of working with movement data, and we emphasize that it will often result in a much deeper understanding of observed patterns, can help to identify optimal analytical approaches for a given data set, and can eventually lead to more meaningful conclusions. A main focus should be to determine what characteristic of the movement is changing in order to choose optimal path-signals representing these changes. Further, the functional relevant time frames at which the observed species moves and potentially changes its behavior needs to be assessed carefully [74, 103]. In general, there are multiple path-signals that are commonly used for certain segmentation methods only. For example, in the literature the penalized contrast method [104] is almost exclusively applied with either the first passage or residence time parameters (e.g., [31, 47, 105]). However, as outlined above (Table 1) there are multiple options for drawing information from the observed trajectory using different path parameters. We suggest that new combinations of path-signals or hybrids of different techniques might lead to valuable insights on movement behavior. For instance, instead of the typically used velocity measures for the BCPA (e.g., persistence velocity; [28]) we chose the net-squared displacement parameter as the in input signal to determine the timing of migratory behaviors in our simulated dataset. Different analytical methods can also be combined in a multi-stage approach where, in a first step, a movement path is segmented using one of the methods for detecting change-points within the movement data (e.g., a time-series approach like BCPA). In a second step, a clustering algorithm could be applied for determining groups of segments with the potentially same behavior (e.g., Step 4 in Fig. 1). In a final step, the segments of the different clusters of movement behavior could be linked to various types of environmental data (e.g., using a step-selection analysis [106, 107]). For example, Zhang et al. [41] applied such a multi-stage approach to determine a number of distinct behaviors within the movement data of little penguins (*Eudyptula minor*) and compared the location and timing of the behavioral switches between the sampled individuals. However, throughout this “top-down” process uncertainties of the chosen segmentation method are potentially projected on to the results of the subsequent analyses which could lead to biased results and interpretations. Currently, it is not clear how severe such uncertainties are for subsequent analyses and ecological inferences.

## Future research needs

The continuing improvement of tracking devices will provide researchers with long-term movement data at high spatial and temporal resolutions [7]. Additionally, the establishment of collaborative projects and data collections will continue to facilitate analyses across many individuals, species, and study areas [4, 108]. To fully realize the potential of this abundant high quality data, powerful analytical techniques are needed. While a substantial variety of methods for path segmentation already exists, we have only just begun to explore the analytical options for path-level movement data, and many more methods will likely be developed in the future. Ideally, these future methods will allow us to quantitatively compare multiple trajectories within and among individuals, so that we can gain a better understanding of the drivers of individual movement paths and underlying behaviors across time and space. For example, this could be accomplished by new topology-based methods using similarity comparisons [109] and pattern recognition [110], as well as data mining of either time-series or the original trajectory data [111, 112].

Future methods should also combine path characteristics with other relevant information such as activity, metabolic and acceleration data [113] or information on body temperature derived from bio-logging devices [114]. Furthermore, the effects of habitat and weather on individual movement behavior could be incorporated into path-level analyses using high resolution environmental and climate data [115, 116].

Regardless of how path segmentation will be improved in the future, a crucial aspect is the evaluation and comparison of available approaches, and the development of guidelines for matching methods to specific research questions. We have provided general suggestions for choosing among methods for three broad types of research questions. However, we feel that it is currently not yet possible to provide a detailed assessment of each of the listed methods we identified for path segmentation (Table 2). For this, it would be necessary to analyze multiple data sets with different characteristics and with different research questions in mind. While suitable data sets for this can probably be identified, we also encourage researchers to make stronger use of individual-based simulations to compare and evaluate segmentation approaches (e.g., [17, 117]). Such validation and accuracy assessment of different methods could also be improved by direct observations [19], via unmanned aerial vehicles [118] (UAVs), or other animal-born logging devices such as video cameras [119, 120].

## Conclusions

Overall, future studies will likely provide a more detailed understanding of the advantages and limitations of different methods for path segmentation. However, given the complexity of segmentation analyses, and considering the variety of research questions that can be addressed with them, it is unlikely that a single method will universally be ‘best’ for all questions and data sets. Hence, while method development and evaluation are clearly crucial, the most important aspect of working with movement data is to define precise research questions [121]. We hope that our overview of currently available segmentation methods provides a first starting point for researchers interested in applying these approaches, so that they can dedicate even more time and energy to defining meaningful questions related to individual movement behavior.

## Additional files

Additional file 1: S1. Overview and detailed description of different methods for path segmentation. (PDF 140 kb) Additional file 2: S2. Detailed illustration of path segmentation approaches. Contains R code for simulating a movement dataset and subsequently applying three different approaches for answering different research questions. (PDF 1519 kb)

## Abbreviations

BCPA: Behavioral Change Point Analysis
BPMM: Bayesian Partitioning of Markov Models
GPS: Global Positioning System
HMM: Hidden Markov Model
NSD: Net-squared displacement
SSM: State-Space Model
UAV: Unmanned Aerial Vehicle
VHF: Very High Frequency (Radio Telemetry)
