# Supplementary material for: Path segmentation for beginners: an overview of current methods for detecting changes in animal movement patterns
Source: Mov Ecol. 2016 Sep 1;4(1):21. doi: 10.1186/s40462-016-0086-5 (PMC5010771; doi:10.1186/s40462-016-0086-5)
Supplement: Additional file 1: S1. — Overview and detailed description of different methods for path segmentation. (PDF 140 kb) [file 40462_2016_86_MOESM1_ESM.pdf]

Edelhoff, Signer & Balkenhol: Path segmentation for beginners: An overview of current methods for detecting changes in animal movement patterns

Supplement S1: Description of individual segmentation methods

Here we provide more detailed information on available methods for path segmentation listed in the publication. Further, we cite literature with applied examples to illustrate the utility of the different methods. Table S1 summarizes basic statistical properties of the discussed methods and lists background papers and availability of code for implementing methods in the program *R* [1].

As outlined in the main article, the presented methods could generally be distinguished based on their analytical background. For a better overview, we assigned the presented methods to three different categories based on whether they focus predominantly on path-topology, or apply different time-series based analyses. Within the latter, one can further distinguish state-space modeling approaches from other general time-series analyses which focus on .

Table S1: Statistical characteristics of the different methodological approaches within the three categories of segmentation methods SI indicates the required sampling interval, which can be either irregular (-), strictly regular (+) or both (-/+). AC provides information on whether a method accounts for (+), neglects (-) or only partly implements (~) estimates of temporal autocorrelation. Further, for each method an outline of the analytical approach, necessary specifications (e.g., parameter settings) and the generated output (results) are listed.

Category	Method	SI	AC	Statistical Approach	Specifications	Result	References	R Implementation
**Topology-Based Analyses**	Thresholding	-/+	-	Indexing/classifying each relocation based on thresholding scheme	Thresholding scheme (absolute or relative threshold values for single or multiple path parameters)	assigned index of each relocation	[2-5]	see Supplement S2
	Change Point Test	-/+	-	Permutation test for detecting significant change points in movement direction between a starting point and an attraction point	subtrajectory defining the potential point of attraction and a starting point; parameters for permutation test	positions of significant change points	[6, 7]	R code provided in [6]
	Spatio-Temporal Criteria Segmentation	-/+	-	Search algorithm for optimal segmentation based on monotone criteria; segment size is increased as long as predefined criteria are fulfilled	monotone criteria (based on path parameter)	positions of significant change points	[8, 9]	NA, theoretical and MATLAB code provided in [8]
	Supervised Classification	-/+	-	Regression trees, vector support machines, random forests, machine learning algorithms; tests for assessing classification accuracy	Multivariate set of path parameters (signals); training set for fitting the classification algorithm	assigned class of each relocation	[10-12]	e.g. *e1071* [13], *tree* [14] packages
	Clustering	-/+	-	K-means Clustering, Expectation-Maximization Binary Clustering; e.g. gap-statistics for determining optimal number of clusters	Multivariate set of path parameters (signals); number of clusters, tolerance value	assigned cluster of each relocation	[15, 16]	*EMbC* [17], *cluster* [18] packages
	Line Simplification	-/+	-	Reduction of the trajectory dimension by removing relocations which do not add to variation e.g. using the Douglas-Peucker Algorithm	tolerance parameter defining the sensitivity for removing a relocation	position of significant change points (i.e., remaining relocations)	[19, 20]	*rgeos* [21] package
	Bayesian Partitioning of Markov Models (BPMM)	+	~	Partitioning and classification algorithm for determining the number and sequence of homogenous processes (models) within a signal (time series) using randomized likelihood estimation	input path signal (time series); number and distributions of candidate models (processes)	estimated state of each relocation and distributions of state sequences	[22-24]	*adehabitatLT* [24] package
**Time-Series Analyses**	Piecewise Regression	-/+	+	Split linear (time) series into most representative segments and fit a polynomial model for each segment	incorporated path signal and model settings	positions of significant change points	[25, 26]	*segmented* [27] package
	Penalized Contrast Method (PCM)	+	~	Non-parametric segmentation of signal; the unknown number of segments is estimated by minimizing a penalized contrast function	input either based on mean, var or mean & var of input signal; minimum segment length; maximum number of change points	positions of significant change points	[28-30]	*adehabitatLT* [24] package
	Behavioral Change Point Analysis (BCPA)	-/+	+	Likelihood-based method for detecting significant change points; applies moving window over continuous autocorrelated time series of input signal	input path signal (time series); window size; sensitivity parameter (BIC restriction); minimum number of change point detections	positions of significant change points	[22, 31]	*bcpa* [32] package
	Pruned Exact Linear Time (PELT) Algorithm	+	~	Pruning search method for detecting optimal number and locations of change points minimizing given cost and linear penalty functions	input path signal; type of expected data changes (mean, var, mean & var); penalty method (SIC, BIC)	positions of significant change points	[33, 34]	*changepoint* [72]
	Behavioral Movement Segmentation (BMS)	+	~	Combined search algorithm using BIC values for optimizing segmentation based on parsimony and subsequent K-means clustering for assigning segments to similar behaviors	minimum segment length, sampling resolution, number of behavioral clusters	path-segments and their behavioral classification	[36]	Mathematical code available from author [36]
**State-Space Models**	Hidden-Markov Models (HMM)	+	+	Expectation-Maximization, maximum likelihood or Bayesian likelihood estimation; Viterbi and Baum-Welch algorithms for estimating state sequences	number of hidden states, input path parameters, assumed parameter distributions	assigned hidden state of each relocation	[37 – 39]	*moveHMM* [40]; R code provided in [41, 42]
	State-Space Models with Location Filtering	-/+	+	Bayesian Likelihood Estimation, Particle Filtering, Kalman Filtering	distributions of path parameters for random walk models, number of states, distributions of switching probabilities (state-space model structure, nr. of particles, weighting scheme)	assigned hidden model of each relocation; estimated parameters of movement model and states	[43-47]	bsam [48], argosTrack [49] packages, R code provided in [49 – 51]

Thresholding

In this most basic segmentation approach, movement modes can be defined directly from observed values of path-signals. Commonly, a set of thresholds is needed as a filtering scheme to separate the relocations into different groups of movement behavior. In many cases, thresholding is used to partition path-signals into either high or low values [37, 51], or to differentiate between localized and long-range movements [2, 52]. The applied thresholds can either be absolute or relative values based on certain observations or hypotheses. The selection of one or more path-signals is mostly based on the research question and data resolution and could be any kind of spatial or temporal property of the movement track (primary or secondary derivatives, see *Table 1* in publication). Further, no data regularity is required in case that signals of relative displacement (e.g. velocity or persistence velocity) are chosen. *Absolute* thresholds usually constitute a cut-off value where a signal is split into two different groups. For example, Zeller et al. [51] defined relocations with a step length less than 200m as "resource use" whereas a step length larger than this threshold was interpreted as actual "movement" (e.g., dispersal). Similarly, Gutenkunst et al. [5] applied a low-pass filter on the ratio between the net-squared displacement and the total length of a movement track of Atlantic bluefin tuna (*Thunnus thynnus*). A predefined threshold of this ratio was used to distinguish localized from long-ranged movements. In contrast to that, *relative* thresholds are often based on the distribution of the considered path-signal, for example by testing whether the observed values are higher or lower than those contained within the 95% confidence interval across all observations [4].

Thresholding can also be extended to multiple path-signals summarized around one or more relocations (e.g., using a moving-window or circular neighborhood) which in the next step are classified according to a thresholding scheme. For example, LaPoint et al. [53] identified potential corridor use behavior of fishers (*Martes pennanti*) based on multiple relocations which were

parallel and comparably linear in direction at a certain speed. To calculate this kind of parallelism of multiple movements, they introduced a path parameter called "pseudo-azimuth" (Table 1 in the main manuscript) which is based on a buffer around midpoints between consecutive relocations.

Supervised Classification

These algorithms have been applied to assign relocations (steps) to different classes of movement behavior based on multiple path-signals. For this, individual steps of a subset of available data (e.g., a training dataset) are assigned to certain classes of movement behavior either visually or by applying a threshold approach as described above. The remaining data sets are then fitted to this classification scheme using either decision trees [54], support vector machines [10] or classification trees [11].

Clustering

Clustering can be regarded as a type of unsupervised classification, where no training data is used to define the groups that the data should be assigned to. In the context of movement data, clustering methods aim to identify distinctive groups within a multivariate set of path-signals without any prior assumptions on the underlying processes [55]. For cluster analyses in general, test statistics have been developed to assess classification accuracy and to find the optimal value for the *k* number of clusters that should be distinguished (e.g. [56, 57]). Further, algorithm performance will depend on the distribution of the parameter values used for clustering (usually one ore multiple path-signals). For example, Van Moorter et al. [55] used a classic *k*-means clustering approach with several parameters including step-length, turning angles and activity data to group movements of elk (*Cervus elaphus*) into within and between feeding patch behaviors. The expectation-maximization binary clustering algorithm (EmBC; [58]) was used by Louzao et al. [59] to distinguish four different behavioral modes in the movements of wandering albatrosses (*Diomedea*

exulans). This method essential splits the relocations into different groups based on a combination of either high or low values for two different path-signals (e.g., speed and turning angles).

Spatio-Temporal Criteria Segmentation

This special type of thresholding relies on a search algorithm that extends an initial segment as long as path-signals at each step fulfill a certain criterion [8, 9]. Thus, the approach essentially attempts to obtain an optimal segmentation of a trajectory, in terms of a minimum number of homogeneous segments. For example, path-signals can be compared to predefined ranges of values such as upper and lower bounds for movement speeds or directional changes that are expected to occur during known movement modes [8]. Consecutive steps are then included in the same segment as long as observed values fall within these bounds, but separated if outside of expected values. In contrast to simple thresholding, spatio-temporal criteria segmentation is based on the concept of monotone criteria, which means that within each segment defined by certain criteria, any subsegment must also fulfill the criteria (e.g., speed and heading within predefined bounds). For example, Buchin et al. [8] applied this algorithm to differ segments of migration flights from stopovers within the trajectories of white-fronted geese (*Anser albifrons*). The monotone criterion for a segment to be determined as migration flight behavior was that all consecutive relocations had to have bounded headings (angles) of around 120°. Therefore, these segments comprised of approximately linear movements. In contrast to that, segments were identified as stopovers when they fulfilled the criterion of containing relocations that where within a disk (radius) of 30km and remained within this disk for a duration of minimum of 48 hours [8]. Finally, the change-points, where the trajectories switched between one of these behavioral states were detected and linked to their recorded timing of the year.

Change Point Test

The method detects significant changes in the observed movement direction or orientation of a

trajectory [6]. For this, a subset of the trajectory based on a potential attraction point (e.g., food source) and the previous relocations back to a starting point (e.g., den or roosting spot) is used as an input. Each of the previous relocations prior to the attraction point is tested “backwards in time” for a change in total direction [6]. The collinearity of the movement vectors before and after a potential change-point are calculated to assess whether movements after a given point are aligned with movements before that point. The significance of the change in directionality is tested using a permutation test, which avoids any assumptions about the distributions of turning angles [6]. The approach is most useful when attraction points can be defined *a priori*. For example, Noser & Byrne [7] applied the change point test to daily travel routes of baboons (*Papio ursinus*) and were able to identify locations where the animals decided to return back towards their sleeping sites, and locations were they adjusted their movements due to important landmarks (e.g., change of topographic slope or tire tracks).

Line Simplification

Line simplification is an approach commonly used in cartography and geographic information science to reduce the number of vertices in geometric objects while maintaining their basic structure [60, 61]. For movement data, this method can be applied to test whether simplifying a trajectory by deleting relocations has a significant impact on the topology of the trajectory. Consecutive relocations that do not change path-topology when being removed can be grouped into the same segment. In contrast, change-points are indicated if their exclusion strongly alters path-topology. As the most prevalent method, the Douglas-Peucker algorithm [61] excludes points which do not add variation along a simplified line between two non-consecutive relocations. For example, Thiebault & Tremblay [20] used this algorithm to segment movement paths of Cape gannets (*Morus capensis*) by calculating the distance between the original path and the simplified, straight line connection of relocations before and after a potential change-point has been removed. If the distance between true

and simplified paths was larger than a specified threshold, a change-point was detected. Since small threshold values lead to small-scale segmentation, and high values to broader-scale segmentation, multiple threshold values should be assessed and compared [20]. The cited example shows that line simplification can also be applied to segment time-ordered data, such that these methods are at the convergence between the two categories of topology-based and time-series analyses.

Bayesian Partitioning of Markov Models (BPMM)

This algorithm can also be interpreted as a hybrid between a method focusing on path-topology on the one hand and accounting for sequential time-series data on the other hand. It is originally derived from a DNA classification method developed by Guéguen [2001] and applies randomized likelihood estimation for determining the optimal number and sequence of a list of candidate Markov models [24]. The input path-signal for the candidate models needs to be ordered in time and derived from a regular trajectory. The input data could be any primary or secondary signal conveying spatial or temporal information (e.g., step length). The candidate models, for example, could have Gaussian distributions with a range of different means while keeping a constant variance [62]. As a result, the trajectory is split into homogeneous segments based on the optimal sequence of Markov models. The BPMM method assumes that the path-signals within these segments are independent, an assumption that is often violated for movement data [62]. Additionally, for each relocation (step) the associated candidate model is specified [24]. Therefore, BPMM has been viewed as a sophisticated classification algorithm (e.g., [62]). However, the list of candidate models could also be interpreted as “hidden states” and therefore we point out that this method could also be potentially applied for identifying hidden processes (see Table 2 of main article).

Methods based on time-series analyses

Piecewise Regression

This approach is also termed “broken-stick” or “segmented” regression and is essentially a type of curve fitting [63]. Basically, the approach finds breakpoints where the relationships between the dependent variable and the independent variable change abruptly. The data are then split at these breakpoints and a separate regression line is fit in each interval. For movement data, the dependent data is a path-signal of interest (e.g., primary or secondary descriptors like net-squared displacement), which is analyzed as a function of time. Detected breakpoints can be interpreted as a change in movement behavior, so that the trajectory can be segmented at that given point in time. For example, Liminana et al. [26] used piecewise linear regression to detect the start of the migratory phase in the movement paths of Montagu’s harriers (*Circus pygargus*). Similar, non-linear types of curve-fitting approaches have been used to determine breaks regarding individual scales of movements [25, 64, 65].

Penalized Contrast Method (PCM)

This method developed by Lavielle [28, 29] as been widely applied in animal movement analyses (e.g., [4, 66]). The optimal number of segments is determined by minimizing a contrast function which rates the differences between signals of the entire trajectory versus the signals of the segmented series. The contrast functions are either based on the mean, standard deviation or a combination of both. The method implies that the contrast function decreases with increasing numbers of segments in the series [29]. In order to avoid visual (and potentially subjective) splitting of the trajectory, Lavielle [28] proposed to use the second derivative of the contrast function and the value at which it reaches a certain threshold. Le Corre et al. [67] used this approach to objectively determine departure and arrival dates in migration patterns of caribou, (*Rangifer tarandus).* In addition, the method requires the definition of the minimum length of resulting segments to avoid over-splitting, and a maximum amount of possible segments in order to limit processing time [24]. PCM is also less susceptible to biases from temporal autocorrelation [29, 66]. The majority of

studies applying the PCM algorithm used either the first-passage- or residence-time (see Table1 of publication) as the input signal (e.g., [68]). However, potentially any primary or secondary signal conveying spatial or temporal information on movement properties (e.g., step length) could be applied.

Behavioral Change Point Analysis (BCPA)

The behavioral change point analysis introduced by Gurarie et al. [31]. consists of several consecutive analytical steps. First, either the persistence or turning velocity is chosen as the input signal as these parameters are less sensitive to irregular sampling (see Table 1 of publication). The signal is modeled as a continuous autocorrelated time-series with three local components (e.g., mean, variance, and temporal autocorrelation). In a second step, the likelihood of a significant change-point within the three local parameters is estimated for a subsample (window) of the time-series [31]. Subsequently, the window is moved forward along the entire time-series. Whether a relocation is a change-point is then evaluated based on a Bayesian Information Criterion (BIC; [69]). which compares different model assumptions ranging from a null-model (no changes) to one, any two, or all three parameters changing at a potential change point. The BCPA does not depend on regular sampling and is able to cope with missing data because primary descriptive features of movements are captured in the velocity signal and the continuous-time modeling framework [31, 70]. However, a set of multiple input parameters, such as the window size and the minimum number of detections of each change-point, have to be set prior to the analysis (see Table S1). For example, Zhang et al. [70] applied the BCPA to detect different behaviors (e.g., foraging) in the movement tracks of little penguins (*Eudyptula minor*).

Pruned Exact Linear Time (PELT) Algorithm

This algorithm searches for an optimal combination of the number and locations of change-points along a time-series [34]. Simply put, the algorithm treats the segmentations produced by different

change-points as competing models, and assesses which model best fits the mean, variance, or a combination of both within the produced segments. Optimality of any set of change-points is defined by a cost function that needs to be minimized and with a penalty term to avoid over-splitting (for example via BIC). Madon & Hingrat [33] used the PELT algorithm to segment movement paths of Macqueen's bustards (*Chlamydotis macqueenii*) and subsequently classified the identified segments into migratory, non-migratory, and staging movements. Similar to the BCPA, the PELT approach is able to detect a set of change-points in an individual movement signal without any *a priori* knowledge on the total number of behavioral modes and switches (see Table S1). Any primary or secondary derivative of path-signals could be used as an input. However, the data is assumed to follow a normal distribution with constant mean, and relocations are assumed to be independent (non-autocorrelated).

Behavioral Movement Segmentation (BMS)

This approach characterizes a behavioral state by a specific mean for one or several path-signals which can be estimated from the data (e.g., any primary or secondary derivative parameter or even acceleration data) [36]. The positions of change-points are also treated as a parameter that can be estimated from the data. The BMS approach attempts to find the most parsimonious set of these two parameters and again uses the BIC [69] to quantify parsimony. For this, a series of different combinations for the number of segments and number of behavioral states is compared and the combination with lowest BIC is chosen. A cluster analysis is then performed to group similar segments and infer behavioral states. One major advantage of the approach is that the estimation of the most likely number and location of behavioral switches can easily be extended to include data other than movement signals. Additionally, the grouping of the resulting segments into clusters of potentially similar movement behaviors is already implemented within the algorithm. For example, Nams [36] combined GPS-relocations and accelerometer (activity) data within the BMS approach

to analyze movement behavior of a fisher (*Martes pennant*). When only using movement speed and turning angles to distinguish behavioral states, four different movement stages could be identified. When additionally accounting for acceleration data, seven behavioral stages could be distinguished, revealing greater complexity in movement behavior than could be inferred from the trajectory alone.

Methods based on state-space models

Methods within this category stem from the broad state-space modeling (SSM) framework. From a statistical perspective, state-space models are special types of time-series analyses also accounting for the correlation structure of consecutive measurements [45]. In general, within this framework the future state of a system is estimated from its previous state(s) through a probabilistic model. For this, two stochastic time-series models, one based on an unobservable state process, and another based on a known observation process are coupled [44, 45]. SSMs differ with regard to the number and composition of the state variables (e.g., discrete vs. continuous), the statistical estimation technique as well as the structure of the main components, the two stochastic process and observation models. Nomenclature for differentiating is unfortunately inconsistent in the literature (e.g., [44, 45, 62, 71]).

In order to provide better guidance for deciding among different SSMs we distinguished three general classes of state-spaces modeling approaches. For example, Hidden Markov models are based on a predefined number of discrete states and typically neglect observation errors within the data. In contrast to that, state-space models can also be extended to include a location filtering component essentially estimating probabilities of different parameters of the movement process including the probable relocations of error-prone movement data. Further, they can work with an undefined number of either discrete or even continuous behavioral states and fit various movement

models (e.g., different variants of a correlated random walk) [22, 45, 71]. Therefore, certain state-space models can also be applied in hierarchical and meta-analyses accounting for individual variations in the number and composition of the hidden states [72–75].

Hidden Markov Models (HMM)

These are special cases of SSMs that estimate properties of a fixed set of discrete hidden states [37, 76]. In an HMM, state transitions are usually driven by first order Markovian processes, which means that a state depends only on the previous state. However, State transitions in HMMs can be modified, so that the switching probability can also depend on several previous states or their durations [42, 77], environmental and social factors [78], as well as habitat data [38, 41]. Some HMMs integrate extensions of the random walk framework (see [79] for more details) as part of their process model [62, 71]. For example, Morales et al. [38] used a Bayesian approach to model movements of elk (*Cervus elaphus*) as a mixture of different random walks, and found that elk movements were either exploratory or encamped, with the latter occurring in open habitats during foraging. Recently, HMMs have also been applied to model behavior based on bio-logger data (e.g., [42, 80]). In summary, the focus of HMM methods is on the estimation of switching probabilities between states, the most likely sequence of the hidden states as well as their length [37, 76]. Parameters in HMMs can be estimated through various statistical techniques, including expectation-maximization [37, 81], likelihood-maximization [42, 76, 82] or Bayesian likelihood estimation [38, 41, 83]. Similarly, several test statistics for evaluating the association between the observed data sequences and the estimated HMM have been suggested, including correct percentage statistics or pseudo-residuals [37, 42, 44].

SSMs with Location Filtering

The majority of SSMs applied in movement-based studies are multi-state random walks integrating

different forms of movement models, such as extensions of the random walk framework, as part of their process component [45, 62]. However, in contrast to HMMs some SSMs do not neglect potential sampling errors of the relocation data but account for them within their observation model structure [44, 71]. Typically, these models include both continuous (e.g., estimated true locations) and one or several discrete behavioral states in the process component [50, 84].

Different Bayesian estimation techniques (e.g., Markov Chain Monte Carlo, MCMC [50]) are usually required since multiple probability distributions need to be integrated along with non-linear structures within the models. Further, MCMC estimation also allows for non-Gaussian error structures in the observation model [44, 45]. Besides MCMC, there are also different so called filtering methods for fitting such SSMs. Filtering methods can be applied to obtain parameters of linear state-space models [71, 85] as well as to estimate the most likely position of missing or biased relocations [86, 87]. For example, Kalman Filtering (KF;[88]) provides unbiased estimates of a first-order autoregressive model (e.g., the diffusion coefficient in a random walk model) given that the movement model is strictly linear with a Gaussian error distribution [89]. Kalman filtering can further account for the influence of environmental covariates [90]. However, the KF is not applicable for estimation of time-varying and discrete behavioral states. Particle Filters (PF) represent Bayesian approaches which overcome those limitations and can also fit non-linear SSMs. They are based on Sequential Monte Carlo sampling using ensembles of random sampling units (particles) which are moved forward by the process model of the state-space framework [45, 71]. The importance of each particle is weighted in order to estimate the likelihood or posterior distributions of the model parameters. PFs are able to estimate non-linear and non-stationary movement models and can also implement non-Gaussian error structures in the process model [47, 89, 91]. Overall, these types of SSMs are highly useful for error-prone or incomplete relocation data with large gaps in sampling frequency. For example, many studies use different SSMs with location filtering in order to account for errors in Argos telemetry data [92–94]. However, all of these

algorithms can be quite complex and computational intensive. More detailed explanations on different statistical options, including model fitting and diagnostics are provided in Jonsen et al. [44]. Different examples for implementations and applications in *R* are presented in [47, 49, 50, 95].

Literature

1. R Core Team: **R: A language and environment for statistical computing**. 2015.

2. Tremblay Y, Roberts AJ, Costa DP: **Fractal landscape method: an alternative approach to measuring area-restricted searching behavior.** *J Exp Biol* 2007, **210**(Pt 6):935–45.

3. Dodge S, Weibel R, Lautenschütz A-K: **Towards a taxonomy of movement patterns**. *Inf Vis* 2008, **7**:240–252.

4. Sur M, Skidmore AK, Exo K-M, Wang T, Ens BJ, Toxopeus AG: **Change detection in animal movement using discrete wavelet analysis**. *Ecol Inform* 2014, **20**:47–57.

5. Gutenkunst R, Newlands N, Lutcavage M, Edelstein-Keshet L: **Inferring resource distributions from Atlantic bluefin tuna movements: an analysis based on net displacement and length of track.** *J Theor Biol* 2007, **245**:243–57.

6. Byrne RW, Noser R, Bates L a., Jupp PE: **How did they get here from there? Detecting changes of direction in terrestrial ranging**. *Anim Behav* 2009, **77**:619–631.

7. Noser R, Byrne RW: **Change point analysis of travel routes reveals novel insights into foraging strategies and cognitive maps of wild baboons**. *Am J Primatol* 2013(MAY 2014).

8. Buchin M, Kruckenberg H, Kölzsch A: **Segmenting Trajectories by Movement States**. In *Advances in Spatial Data Handling, Geospatial Dynamics, Geosimulation and Exploratory Visualization*. Edited by Timpf S, Laube P. Berlin, Heidelberg: Springer Berlin Heidelberg; 2013:15–25. [*Advances in Geographic Information Science*]

9. Buchin M, Driemel A, Van Kreveld M, Sacristan V: **Segmenting trajectories: A framework and algorithms using spatiotemporal criteria**. *J Spat Inf Sci* 2011, **3**:33–63.

10. Dodge S, Weibel R, Forootan E: **Revealing the physics of movement: comparing the similarity of movement characteristics of different types of moving objects**. 2009:419–434.

11. Shamoun-Baranes J, Bom R, van Loon EE, Ens BJ, Oosterbeek K, Bouten W: **From sensor data to animal behaviour: an oystercatcher example.** *PLoS One* 2012, **7**:e37997.

12. Soleymani A, Cachat J, Robinson K: **Integrating cross-scale analysis in the spatial and temporal domains for classification of behavioral movement**. *J Spat Inf Sci* 2014.

13. Dimitriadou E, Hornik K, Leisch F, Meyer D, Weingessel A: **e1071: Misc Functions of the**

Department of Statistics, Probability Theory Group (Formerly: E1071), TU Wien. 2015.

14. Ripley B: **tree: classification and regression trees. R package version 1.0-36**. 2015.

15. Van Moorter B, Visscher DR, Jerde CL, Frair JL, Merrill EH: **Identifying Movement States From Location Data Using Cluster Analysis**. *J Wildl Manage* 2010, **74**:588–594.

16. Garriga J, Palmer JR, Oltra A, Bartumeus F, Vall C: **Expectation-Maximization Binary Clustering for Behavioural Annotation**. 2015.

17. Garriga J, Bartumeus F: **EMbC: Expectation-Maximization Binary Clustering. R package version 1.9.3.** 2015

18. Maechler M, Rousseeuw P, Struyf A, Hubert M, Hornik K, Studer M, Roudier P: **cluster: “Finding Groups in Data”: Cluster Analysis Extended. R package version 2.0.3**. 2015.

19. Douglas D, Peucker T: **Algorithms for the reduction of the number of points required to represent a digitized line or its caricature**. *Cannadian Cartogr* 1973, **10**:1973.

20. Thiebault A, Tremblay Y: **Splitting animal trajectories into fine-scale behaviorally consistent movement units: breaking points relate to external stimuli in a foraging seabird**. *Behav Ecol Sociobiol* 2013, **67**:1013–1026.

21. Bivand R, Rundel C, Pebesma E, Hufthammer KO: **rgeos: Interface to Geometry Engine - Open Source (GEOS). R package version 0.3-15**. 2015.

22. Gurarie E, Bracis C, Delgado M, Meckley TD, Kojola I, Wagner CM: **What is the animal doing? Tools for exploring behavioral structure in animal movements**. *J Anim Ecol* 2016, **85**:69–84.

23. Gueguen L: **Segmentation by maximal predictive partitioning according to composition biases**. *Comput Biol* 2001.

24. Calenge C: **Analysis of Animal Movements in R: the adehabitatLT Package**. 2011:1–85.

25. Johnson C, Parker K, Heard D, Gillingham M: **Movement parameters of ungulates and scale-specific responses to the environment**. *J Anim Ecol* 2002, **71**:225–235.

26. Liminana R, Soutullo A, Urios V: **Autumn migration of Montagu’s harriers Circus pygargus tracked by satellite telemetry .** *J Ornithol* 2007, **148**:517–523.

27. Muggeo VMR: **segmented: Regression Models with Breakpoints/Changepoints Estimation. R package version 0.5-1.4**. 2015.

28. Lavielle M: **Using penalized contrasts for the change-point problem**. *Signal Processing* 2005, **85**:1501–1510.

29. Lavielle M: **Detection of multiple changes in a sequence of dependent variables**. *Stoch Process their Appl* 1999, **83**:79–102.

30. Le Corre M, Dussault C, Côté SD: **Detecting changes in the annual movements of terrestrial**

migratory species: using the first-passage time to document the spring migration of caribou. *Mov Ecol* 2014, **2**:19.

31. Gurarie E, Andrews RD, Laidre KL: **A novel method for identifying behavioural changes in animal movement data**. *Ecol Lett* 2009, **12**:395–408.

32. Gurarie E: **bcpa: Behavioral change point analysis of animal movement. R package version 1.1**. 2015.

33. Madon B, Hingrat Y: **Deciphering behavioral changes in animal movement with a “multiple change point algorithm- classification tree” framework**. *Front Ecol Evol* 2014, **2**(July):1–9.

34. Killick R, Fearnhead P, Eckley I a.: **Optimal detection of changepoints with a linear computational cost**. 2011:37.

35. Killick R, Haynes KJ, Eckley IA, Fearnhead P, Lee J: **changepoint: Methods for Changepoint Detection. R package version 2.2**. 2015.

36. Nams VO: **Combining animal movements and behavioural data to detect behavioural states**. *Ecol Lett* 2014:1228–1237.

37. Franke A, Caelli T, Hudson RJ: **Analysis of movements and behavior of caribou (Rangifer tarandus) using hidden Markov models**. *Ecol Modell* 2004, **173**:259–270.

38. Morales J, Haydon D, Frair J: **Extracting more out of relocation data: building movement models as mixtures of random walks**. *Ecology* 2004, **85**:2436–2445.

39. Langrock R, Zucchini W: **Hidden Markov models with arbitrary state dwell-time distributions**. *Comput Stat Data Anal* 2011, **55**:715–724.

40. Michelot T, Langrock R, Patterson T, Rexstad E: **moveHMM: Animal movement modelling using Hidden Markov Models. R package version 1.1**. 2015.

41. Beyer HL, Morales JM, Murray D, Fortin M-J: **The effectiveness of Bayesian state-space models for estimating behavioural states from movement paths**. *Methods Ecol Evol* 2013, **4**:433–441.

42. Patterson T a, Basson M, Bravington M V, Gunn JS: **Classifying movement behaviour in relation to environmental conditions using hidden Markov models.** *J Anim Ecol* 2009, **78**:1113–23.

43. Breed G a., Costa DP, Jonsen ID, Robinson PW, Mills-Flemming J: **State-space methods for more completely capturing behavioral dynamics from animal tracks**. *Ecol Modell* 2012, **235-236**:49–58.

44. Jonsen ID, Basson M, Bestley S, Bravington MV, Patterson T a., Pedersen MW, Thomson R, Thygesen UH, Wotherspoon SJ: **State-space models for bio-loggers: A methodological road map**. *Deep Sea Res Part II Top Stud Oceanogr* 2013, **88-89**:34–46.

45. Patterson T a, Thomas L, Wilcox C, Ovaskainen O, Matthiopoulos J: **State-space models of**

individual animal movement. *Trends Ecol Evol* 2008, **23**:87–94.

46. Forester J, Ives A, Turner M, Anderson D, Fortin D, Beyer H, Smith D, Boyce M: **State-space models link elk movement patterns to landscape characteristics in Yellowstone National Park**. *Ecol Monogr* 2007, **77**:285–299.

47. Dowd M, Joy R: **Estimating behavioral parameters in animal movement models using a state-augmented particle filter.** *Ecology* 2011, **92**:568–75.

48. Jonsen I: **bsam: Bayesian State-space Models for Animal Movement. R Package Version 0.43-1.** 2014.

49. Albertsen CM, Whoriskey K, Yurkowski D, Nielsen A, Flemming JM: **Fast fitting of non-Gaussian state-space models to animal movement data via Template Model Builder**. *Ecology* 2015, **96**:2598–2604.

50. Jonsen ID, Flemming J, Myers R: **Robust State-Space Modeling of Animal Movement Data**. *Ecology* 2005, **86**:2874–2880.

51. Zeller KA, McGarigal K, Cushman SA, Beier P, Vickers TW, Boyce WM: **Using step and path selection functions for estimating resistance to movement: pumas as a case study**. *Landsc Ecol* 2015.

52. Dzialak MR, Olson C V, Webb SL, Harju SM, Winstead JB: **Incorporating within- and between-patch resource selection in identification of critical habitat for brood-rearing greater sage-grouse**. *Ecol Process* 2015, **4**.

53. LaPoint S, Gallery P, Wikelski M, Kays R: **Animal behavior, cost-based corridor models, and real corridors**. *Landsc Ecol* 2013.

54. Soleymani A, Loon E van, Weibel R: **Capability of movement features extracted from GPS trajectories for the classification of fine-grained behaviors**. *agile-online.org* 2014:3–6.

55. Van Moorter B, Visscher DR, Jerde CL, Frair JL, Merrill EH: **Identifying Movement States From Location Data Using Cluster Analysis**. *J Wildl Manage* 2010, **74**:588–594.

56. Steinley D: **K-means clustering: a half-century synthesis**. *Br J Math Stat.* 2006.

57. Legendre P, Legendre L: **Numerical ecology**. 2012.

58. Garriga J, Palmer J, Oltra A, Bartumeus F: **Expectation-Maximization Binary Clustering for Behavioural Annotation**. *arXiv arXiv:1503.04059v1.* 2015.

59. Louzao M, Wiegand T, Bartumeus F, Weimerskirch H: **Coupling instantaneous energy-budget models and behavioural mode analysis to estimate optimal foraging strategy: an example with wandering albatrosses**. *Mov Ecol* 2014, **2**:8.

60. Saalfeld A: **Topologically Consistent Line Simplification with the Douglas-Peucker Algorithm**. *Cartogr Geogr Inf Sci* 1999, **26**:7–18.

61. Douglas D, Peucker T: **Algorithms for the reduction of the number of points required to represent a digitized line or its caricature**. *Cartogr Int J …* 1973, **10**:1973.

62. Gurarie E, Bracis C, Delgado M, Meckley TD, Kojola I, Wagner CM: **What is the animal doing? Tools for exploring behavioral structure in animal movements**. *J Anim Ecol* 2015

63. Neter J, Wasserman W, Kutner M: **Applied linear statistical models: Regression, analysis of variance, and experimental designs, Homewood, IL: Richard D. Irwin**. *Inc[701]* 1985.

64. Sibly RM, Nott HMR, Fletcher DJ: **Splitting behaviour into bouts**. *Anim Behav* 1990, **39**:63–69.

65. Saher DJ, Schmiegelow FK a: **Movement pathways and habitat selection by woodland caribou during spring migration**. *Rangifer* 2005, **16**:143–154.

66. Barraquand F, Benhamou S: **Animal movements in heterogeneous landscapes: identifying profitable places and homogeneous movement bouts.** *Ecology* 2008, **89**:3336–48.

67. Le Corre M, Dussault C, Côté SD: **Detecting changes in the annual movements of terrestrial migratory species: using the first-passage time to document the spring migration of caribou**. *Mov Ecol* 2014, **2**:1–11.

68. Sommerfeld J, Kato A, Ropert-Coudert Y, Garthe S, Hindell M a: **Foraging parameters influencing the detection and interpretation of area-restricted search behaviour in marine predators: a case study with the masked booby.** *PLoS One* 2013, **8**:e63742.

69. Burnham K, Anderson D: **Model selection and multimodel inference: a practical information-theoretic approach**. 2002.

70. Zhang J, O'Reilly KM, Perry GLW, Taylor G a., Dennis TE: **Extending the Functionality of Behavioural Change-Point Analysis with k-Means Clustering: A Case Study with the Little Penguin (Eudyptula minor)**. *PLoS One* 2015, **10**:e0122811.

71. Patterson TA, Parton A, Langrock R, Blackwell PG, Thomas L, King R, King R: **Statistical modelling of animal movement: a myopic review and a discussion of good practice**. *arXiv Prepr* 2016, **arXiv:1603**.

72. Jonsen I, Myers R, Flemming J: **Meta-analysis of animal movement using state-space models**. *Ecology* 2003, **84**:3055–3063.

73. Jonsen ID, Myers RA, James MC: **Robust hierarchical state-space models reveal diel variation in travel rates of migrating leatherback turtles**. *J Anim Ecol* 2006, **75**:1046–1057.

74. Eckert S, Moore J: **Modeling loggerhead turtle movement in the Mediterranean: importance of body size and oceanography**. *Ecol Appl* 2008, **18**:290–308.

75. Mills Flemming J, Jonsen ID, Myers R a., Field C a.: **Hierarchical State-Space Estimation of leatherback turtle navigation ability**. *PLoS One* 2010, **5**.

76. Zucchini W, Raubenheimer D, MacDonald I: **Modeling time series of animal behavior by**

means of a latent-state model with feedback. *Biometrics* 2008, **64**:807–815.

77. Langrock R, King R, Matthiopoulos J: **Flexible and practical modeling of animal telemetry data: hidden Markov models and extensions**. *Ecology* 2012, **93**:2336–2342.

78. Bergman CM, Fryxell JM, Gates CC, Fortin D: **Ungulate foraging strategies: energy maximizing or time minimizing?** *J Anim Ecol* 2008, **70**:289–300.

79. Codling E a, Plank MJ, Benhamou S: **Random walk models in biology.** *J R Soc Interface* 2008, **5**:813–34.

80. Dean B, Freeman R, Kirk H, Leonard K, Phillips R a., Perrins CM, Guilford T: **Behavioural mapping of a pelagic seabird: combining multiple sensors and a hidden Markov model reveals the distribution of at-sea behaviour**. 2013.

81. Rabiner LR: **A tutorial on hidden Markov models and selected applications in speech recognition**. *Proc IEEE* 1989, **77**:257–286.

82. Langrock R, Kneib T, Sohn A, DeRuiter SL: **Nonparametric inference in hidden Markov models using P-splines.** *Biometrics* 2015, **71**:520–8.

83. Roberts S, Guilford T, Rezek I, Biro D: **Positional entropy during pigeon homing I: application of Bayesian latent state modelling.** *J Theor Biol* 2004, **227**:39–50.

84. Hopcraft JGC, Morales JM, Beyer HL, Borner M, Mwangomo E, Sinclair a. RE, Olff H, Haydon DT: **Competition, predation, and migration: Individual choice patterns of Serengeti migrants captured by hierarchical models**. *Ecol Monogr* 2014, **84**:355–372.

85. Anderson-Sprecher R, Ledolter J: **State-Space Analysis of Wildlife Telemetry Data**. *J Am Stat Assoc* 2012.

86. Sibert JR, Musyl MK, Brill RW: **Horizontal movements of bigeye tuna (Thunnus obesus) near Hawaii determined by Kalman filter analysis of archival tagging data**. *Fish Oceanogr* 2003, **12**(September 2001):141–151.

87. Austin D, McMillan JI, Bowen WD: **a Three-Stage Algorithm for Filtering Erroneous Argos Satellite Locations**. *Mar Mammal Sci* 2003, **19**:371–383.

88. Kalman RE, Bucy RS: **New results in linear filtering and prediction theory**. *J Basic Eng* 1961, **83**:95–108.

89. Royer F, Fromentin J, Gaspar P: **A state–space model to derive bluefin tuna movement and habitat from archival tags**. *Oikos* 2005, **3**(November 2004):473–484.

90. Forester JD, Ives AR, Turner MG, Anderson DP, Fortin D, Beyer HL, Smith DW, Boyce MS: **State–Space Models Link Elk Movement Patterns To Landscape Characteristics in Yellowstone National Park**. *Ecol Monogr* 2007, **77**:285–299.

91. Andersen KH, Nielsen a, Thygesen UH, Hinrichsen H-H, Neuenfeldt S: **Using the particle filter to geolocate Atlantic cod (** ***Gadus morhua*** **) in the Baltic Sea, with special emphasis on**

determining uncertainty. *Can J Fish Aquat Sci* 2007, **64**:618–627.

92. Patterson T a., McConnell BJ, Fedak M a., Bravington M V., Hindell M a.: **Using GPS data to evaluate the accuracy of state-space methods for correction of Argos satellite telemetry error**. *Ecology* 2010, **91**:273–285.

93. Costa DP, Robinson PW, Arnould JPY, Harrison A-L, Simmons SE, Hassrick JL, Hoskins AJ, Kirkman SP, Oosthuizen H, Villegas-Amtmann S, Crocker DE: **Accuracy of ARGOS locations of Pinnipeds at-sea estimated using Fastloc GPS.** *PLoS One* 2010, **5**:e8677.

94. Silva M a, Jonsen I, Russell DJF, Prieto R, Thompson D, Baumgartner MF: **Assessing performance of bayesian state-space models fit to argos satellite telemetry locations processed with kalman filtering.** *PLoS One* 2014, **9**:e92277.

95. Pedersen MW, Berg CW, Thygesen UH, Nielsen a., Madsen H: **Estimation methods for nonlinear state-space models in ecology**. *Ecol Modell* 2011, **222**:1394–1400.
