# Supplementary material for: Path segmentation for beginners: an overview of current methods for detecting changes in animal movement patterns
Source: Mov Ecol. 2016 Sep 1;4(1):21. doi: 10.1186/s40462-016-0086-5 (PMC5010771; doi:10.1186/s40462-016-0086-5)
Supplement: Additional file 2: S2. — Detailed illustration of path segmentation approaches. Contains R code for simulating a movement dataset and subsequently applying three different approaches for answering different research questions. (PDF 1519 kb) [file 40462_2016_86_MOESM2_ESM.pdf]

Supplement S2: Applied Illustration of Path Segmentation Approaches

Edelhoff, Signer & Balkenhol

Aim of this supplement

In this supplement we illustrate the basic approach of how to segement a path. We simulate tracking data, go through explorative steps, and then apply three different segementation methods.

Set up

```
library(secr)
library(raster)
library(moveHMM)
library(adehabitatLT)
library(lubridate)
library(bcpa)

set.seed(12322211)

## Utility function
genHabitat <- function(nc, p = 0.1, A = 0.1) {
  r <- make.mask(nx = nc, ny = nc, spacing = 1)
  h <- randomHabitat(r, p = p, A = A)
  r <- raster(xmn=0, xmx=nc, ymn=0, ymx=nc, ncols=nc, nrows=nc)
  r <- rasterize(data.frame(h), r, field=1, background=0)
  r <- as.matrix(r)
  r <- list(hab = r, p = p, A = A, nc = nc)
  class(r) <- c("hab", "matrix")
  r
}
```

Data generation

We use a simple individual based simulation model for 12 months with 24 relocations per day. The movement of the animal was influenced by the time of the day, habitat and attraction to a temporally varying home range center. Turning angles where uniformily distributed between $-pi$ and pi, and step lengths where drawn from a Gamma distribution with scale and shape of 2.9 and 0.9 for habitat and 1.2 and 5 for the matrix respectively Animals moved with a probability of 0.3 during the night and a probability of 1 during the day. At time step t, the animal chose 20 candidate locations where to move at time step $t + 1$. One relocation was randomly chose with probably ω. Where ω is the sum of the transformed distance to both home range centers. We transformed the distances using the exponential probability density function. Coefficients for the probability of movement and the the attraction to either home range center varied over time, while the coeffiecient for habitat was constant.

```r
n_months <- 12
n <- 24 * 30 * n_months  # one relocation every hour

pm_day <- 1
pm_night <- 0.3

b0 <- rep(c(rep(pm_night, 5), seq(pm_night, pm_day, length.out = 2), rep(pm_day, 10),
            seq(pm_day, pm_night, length.out = 2), rep(pm_night, 5)), 30 * n_months)

b1 <- rep(c(1, 0, 1), each = n/3) * 1
b2 <- as.numeric(b1 == 0) * 1

b3 <- 10 # hab matrix

xy0 <- c(100, 100)

x_c1 <- 100
y_c1 <- 100

x_c2 <- 300
y_c2 <- 300

tpl <- raster(xmn = 0, xmx = 400, ymn = 0, ymx = 400, res = 1)
hab <- raster(genHabitat(400, A = 0.5, p = 0.5)$hab, template = tpl)
hab0 <- hab
hab <- hab0
hab[] <- ifelse(hab[] == 0, 1, 2)

set.seed(2090160703)
xy <- matrix(NA, nrow = n, ncol = 2)
xy[1, ] <- xy0


for (i in 2:n) {
  # cand locations
  if (runif(1) < b0[i]) {
    ta <- runif(20, -pi, pi)

    if (raster::extract(hab, xy[i-1, ,drop = FALSE]) == 2) {
      scl <- 2.9
      shp <- 0.9
    } else {
      scl <- 1.2
      shp <- 5
    }
    slen <- rgamma(20, scale = scl, shape = shp)
    x1 <- xy[i - 1, 1] + (cos(ta) * slen)
    y1 <- xy[i - 1, 2] + (sin(ta) * slen)

    d1 <- sqrt((x1 - x_c1)^2 + (y1 - y_c1)^2)
    d2 <- sqrt((x1 - x_c2)^2 + (y1 - y_c2)^2)
    w <- (dexp(d1, rate = 1/25) * b1[i] +
            dexp(d2, rate = 1/25) * b2[i])
```

```
    w[is.na(w)] <- 0
    w <- sample(20, 1, prob = w)
    xy[i, ] <- c(x1[w], y1[w])
  } else {
    xy[i, ] <- xy[i-1, ]
  }
}

xy[, 1] <- xy[, 1] + runif(nrow(xy), -0.1, 0.1)
xy[, 2] <- xy[, 2] + runif(nrow(xy), -0.1, 0.1)
xy <- xy[, 1:2]
xy <- data.frame(xy)
names(xy) <- c("x", "y")
xy$time <- ymd_hm("2000-01-01 00:00") + hours(0:(n-1))
xy$hour <- rep(1:24, n/24) # hour of the day
xy$id <- 1:nrow(xy)
xy$hab <- raster::extract(hab, xy[, 1:2])
```

The simulated data:

```
plot(xy[, 1:2], pch = 20, xlab = "", ylab = "", las = 1, type = "l",
     lwd = 0.5, asp = 1)
```

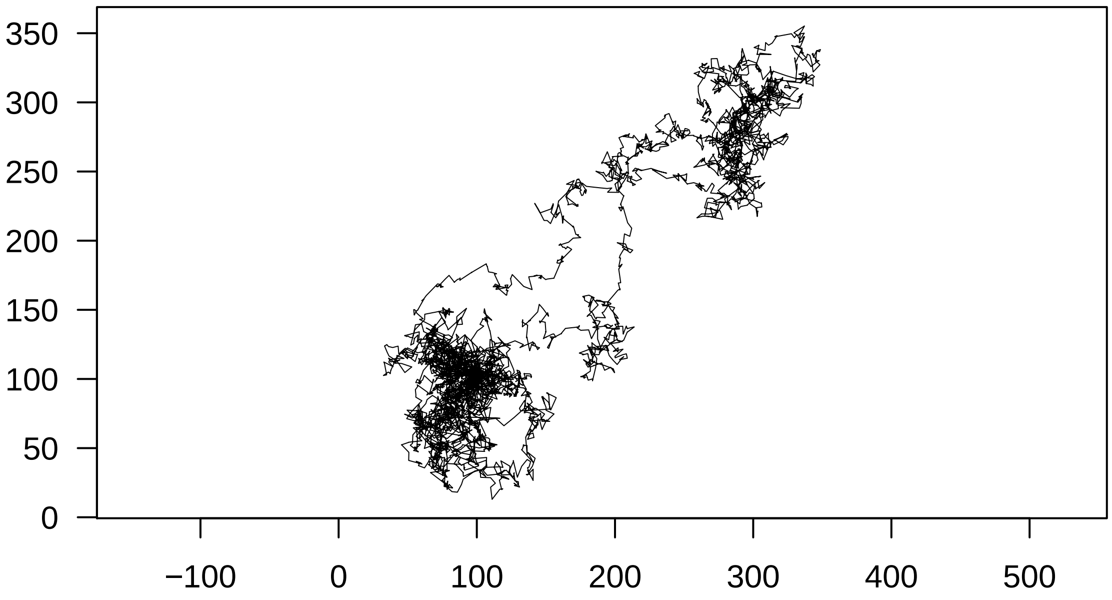

Simulated movement path and habitat composition:

```
plot(hab)
lines(xy[,1:2])
```

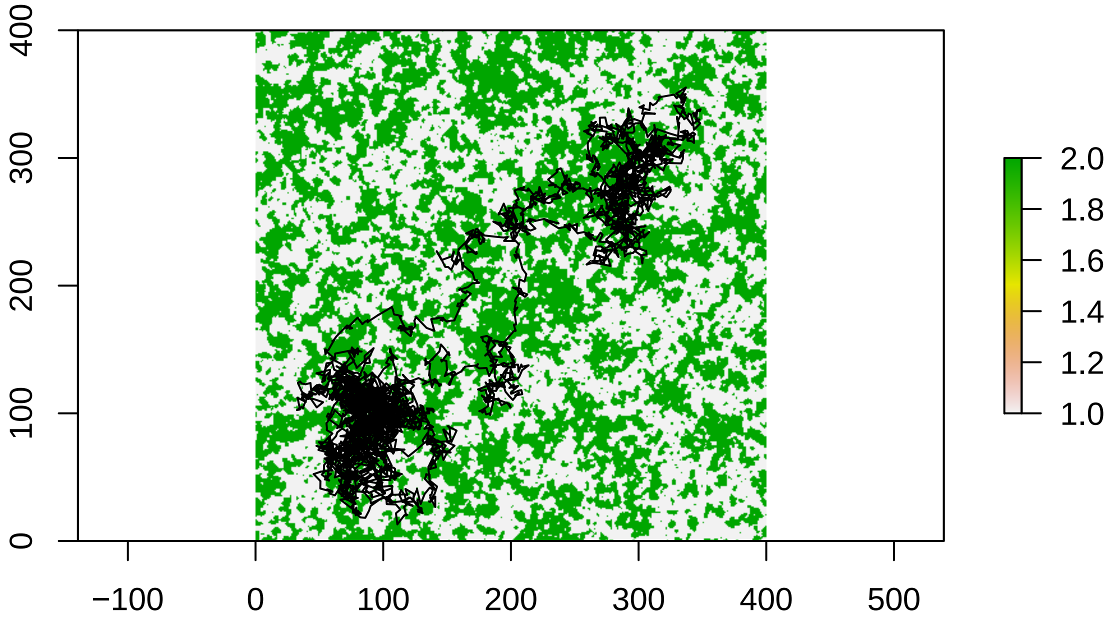

Path metrics

As a next step we calculate and inspect three path metrics. Namely, the step length, the net squared displacement and finally the turning anlges.

```
xy$sl <- c(NA, with(xy, sqrt((head(x, -1) - tail(x, -1))^2 +
                               (head(y, -1) - tail(y, -1))^2)))
xy$nsd <- with(xy, sqrt((x - xy0[1])^2 + (y - xy0[2])^2))
dat <- prepData(xy[, c("x", "y", "hour", "nsd", "hab")], type = "UTM")
```

Plotting path metrics as time series and histograms. First we look at the signals for 1 week.

```
par(mfrow = c(3, 2))
with(dat[1:(24 * 7), ], plot(step, type = "l"))
with(dat[1:(24 * 7), ], hist(step))
with(dat[1:(24 * 7), ], plot(angle, type = "l"))
with(dat[1:(24 * 7), ], hist(angle))
with(dat[1:(24 * 7), ], plot(nsd, type = "l"))
with(dat[1:(24 * 7), ], hist(nsd))
```

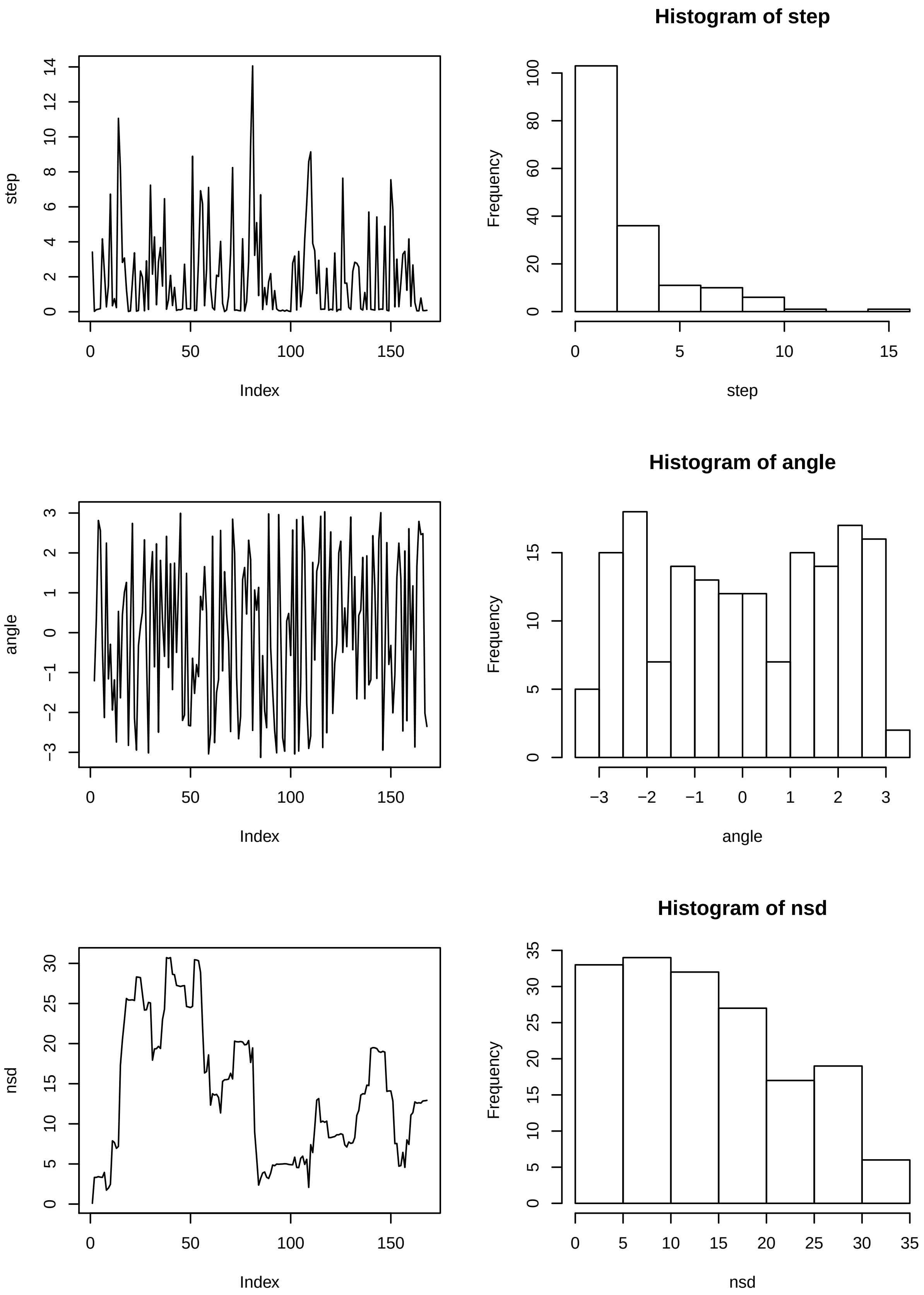

Next we look at the path signals for a whole year.

```r
par(mfrow = c(3, 2))
with(dat, plot(step, type = "l"))
with(dat, hist(step))
with(dat, plot(angle, type = "l"))
with(dat, hist(angle))
with(dat, plot(nsd, type = "l"))
with(dat, hist(nsd))
```

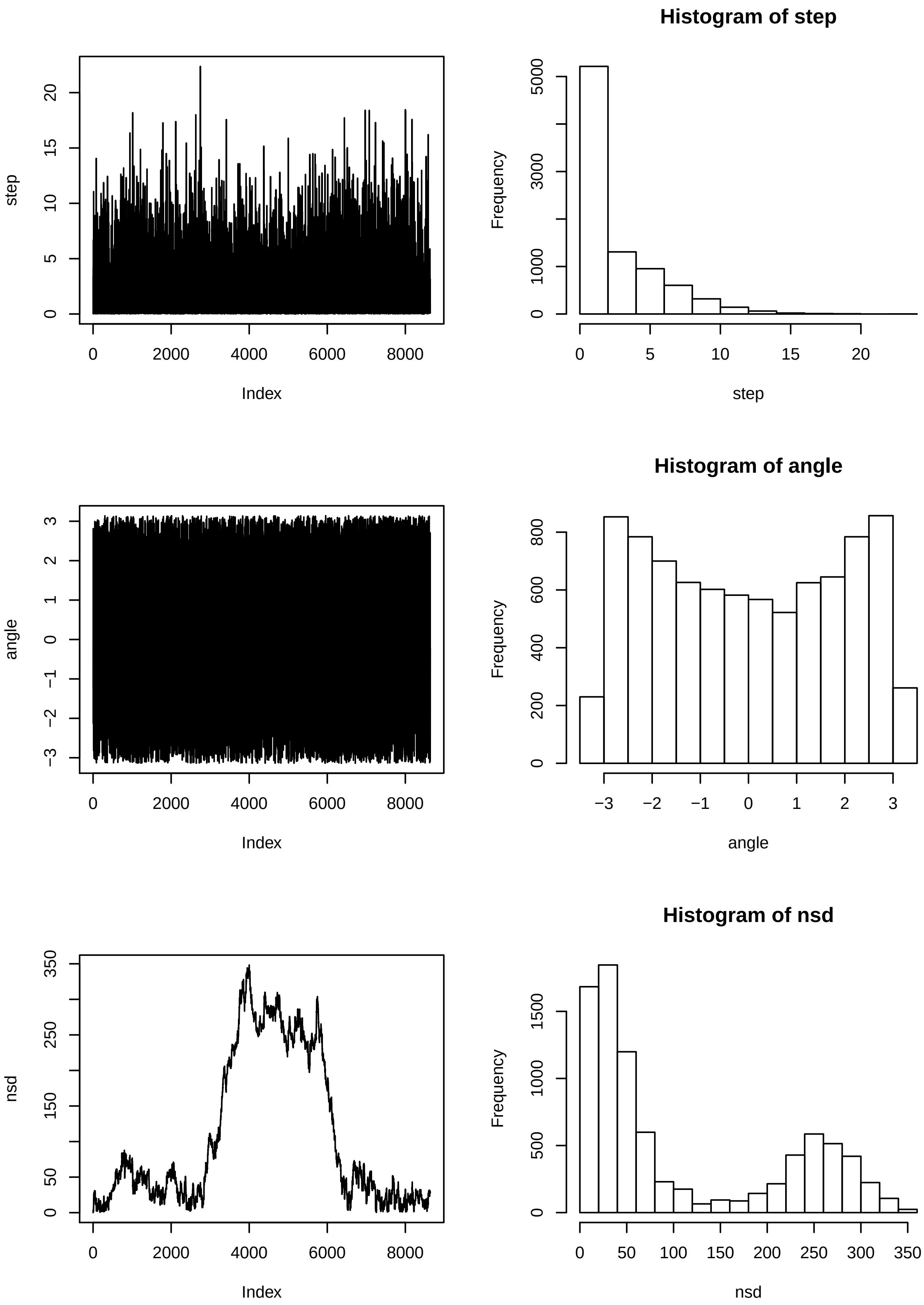

Note, that when plotting path signals for one week we can observe diurnal patterns, while when plotting the

signals for one year we observe a migratory pattern, especially for the net-squared displacement.

Finally, we can look at the same path signal, but as a funciton of different covariates (here habitat and matrix, left column; hour of the day right column).

```
par(mfrow = c(3, 2))
with(dat, boxplot(step ~ hab, main = "step length", xaxt = "n"))
axis(1, at = 1:2, labels = c("matrix", "habitat"))
with(dat, boxplot(step ~ hour, main = "step length", xlab = "hour of the day"))
with(dat, boxplot(angle ~ hab, main = "turning angles", xaxt = "n"))
axis(1, at = 1:2, labels = c("matrix", "habitat"))
with(dat, boxplot(angle ~ hour, main = "turning angles", xlab = "hour of the day"))
with(dat, boxplot(nsd ~ hab, main = "nsd", xaxt = "n"))
axis(1, at = 1:2, labels = c("matrix", "habitat"))
with(dat, boxplot(nsd ~ hour, main = "nsd", xlab = "hour of the day"))
```

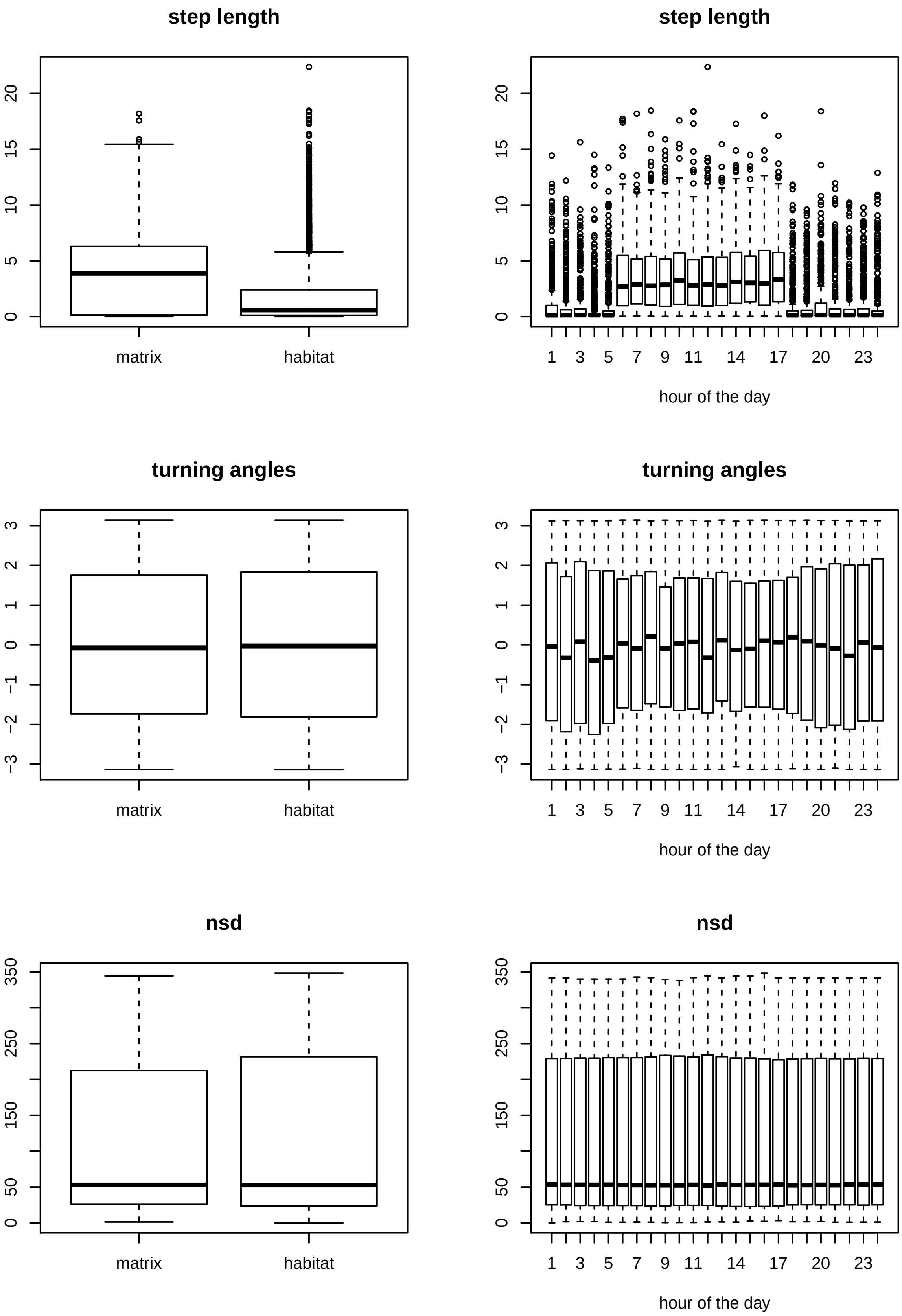
step length
0
5
10
15
20
matrix
habitat
step length
1
3
5
7
9
11
14
17
20
23
hour of the day
turning angles
−3
−2
−1
0
1
2
3
matrix
habitat
turning angles
1
3
5
7
9
11
14
17
20
23
hour of the day
nsd
0
50
150
250
350
matrix
habitat
nsd
1
3
5
7
9
11
14
17
20
23
hour of the day

```r
par(mfrow = c(1, 1))
```

Explorative analyses

First we check if the data is regularly sampled:

```r
table(diff(xy$time))
```

```
##
##    1
## 8639
```

All of the 8639 relocations have a time lag of 1 hour. Therefore, the data has a regular sampling regime.

Next we test for independence in the consecutive path-signals:

```r
# Wald-Wolfowitz Test of Randomness

wawotest(dat$step)
```

```
## 1 NA removed
```

```
##          a         ea         va         za          p
## 1984.61369   -1.00000 8632.54925   21.37102    0.00000
```

```r
wawotest(dat$nsd)
```

```
##          a         ea         va         za          p
## 8636.12915   -1.00000 8637.10049   92.93631    0.00000
```

```r
# correlogram for angular and linear descriptors of a movement path

ind <- acfdist.ltraj(as.ltraj(xy[, 1:2], date = xy$time, id = "a1"), lag = 5)
```

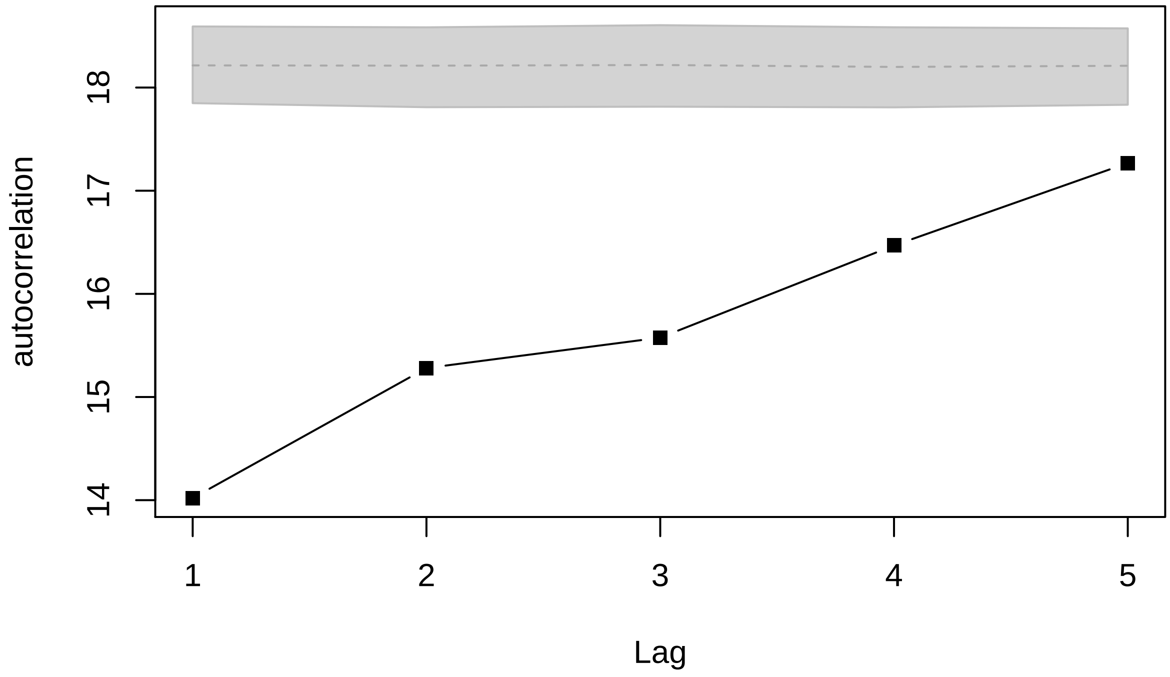


```
ind
```

```
## [[1]]
##          lag.1    lag.2    lag.3    lag.4    lag.5
## obs   14.02014 15.28050 15.57561 16.46903 17.26840
## 2.5%  17.84895 17.80956 17.81445 17.80819 17.83331
## 50%   18.21509 18.21207 18.21890 18.20008 18.21123
## 97.5% 18.59233 18.58381 18.60474 18.58432 18.57373
```

```
ind2 <- acfang.ltraj(as.ltraj(xy[, 1:2], date = xy$time, id = "a1"), lag = 5)
```

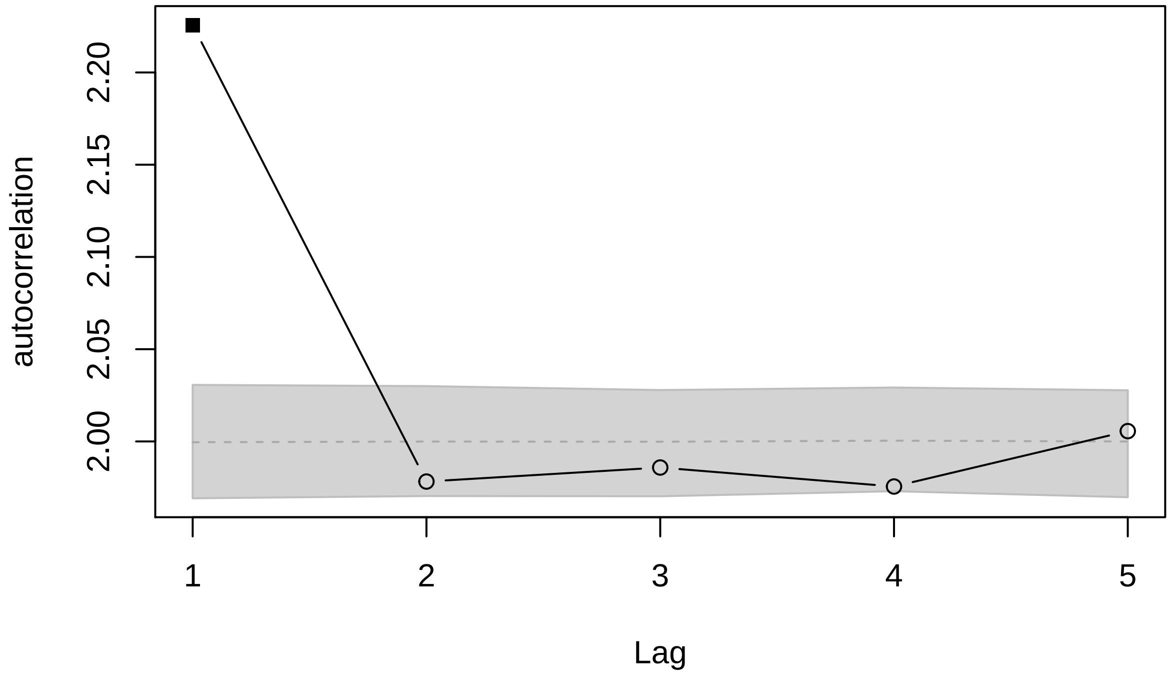

```
ind2
```

```
## [[1]]
##          lag.1    lag.2    lag.3    lag.4    lag.5
## obs   2.225695 1.978259 1.985859 1.975575 2.005591
## 2.5%  1.969194 1.970401 1.970287 1.973024 1.969762
## 50%   1.999576 1.999937 1.999834 2.000412 1.999972
## 97.5% 2.030653 2.029986 2.027849 2.029211 2.027733
```

both tests suggest for correlated data structures.

Path segmentation

We consider three methods: 1. Thresholding, 2. Behavioural Change Point Analysis, and 3. Hidden Markov Models:

Threshholding

A ecologist may assume that due to biological reasoning a step length threshhold of 2 is indicating an important biological phenomena.

```
hist(xy$sl, main="Histogram of Step Length", xlab="Step Length")
abline(v=2, col="red", lwd=3)
```

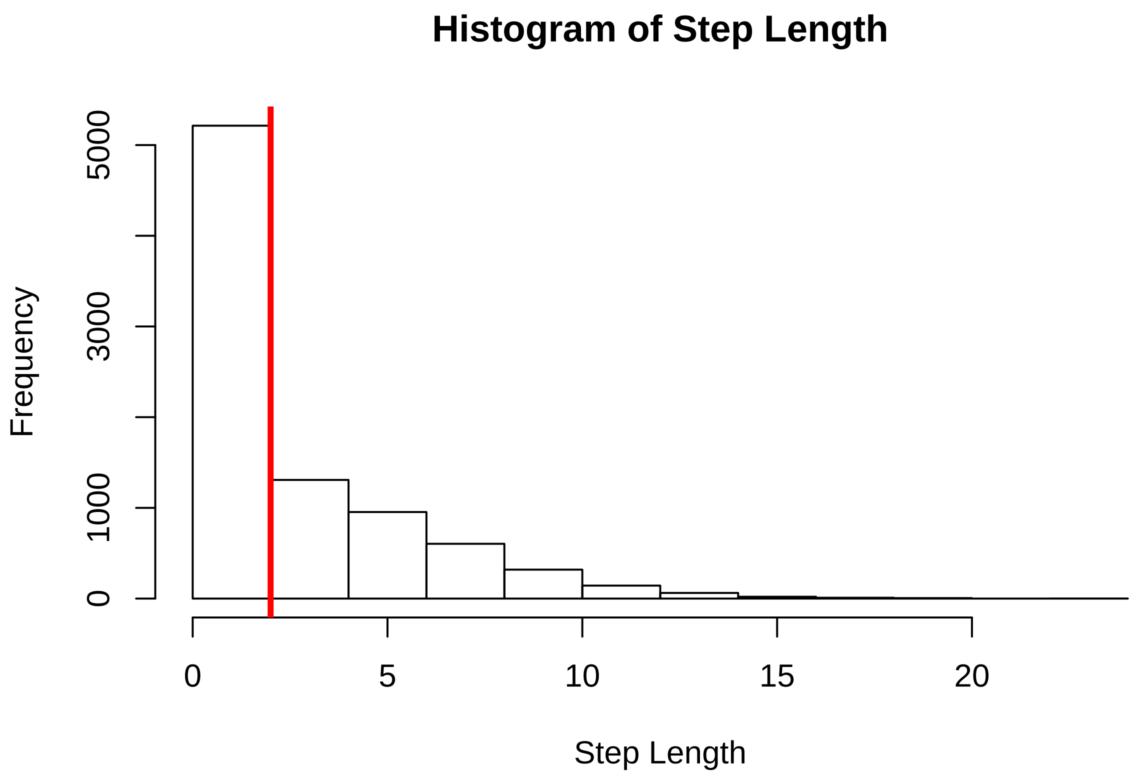

This threshold can than be plotted against the habitat type.

```r
tb <- table(xy$sl < 2, xy$hab)
row.names(tb) <- c("step length >= 2", "step length < 2")
colnames(tb) <- c("matrix", "habitat")
mosaicplot(tb , main = "Treshholding")
```

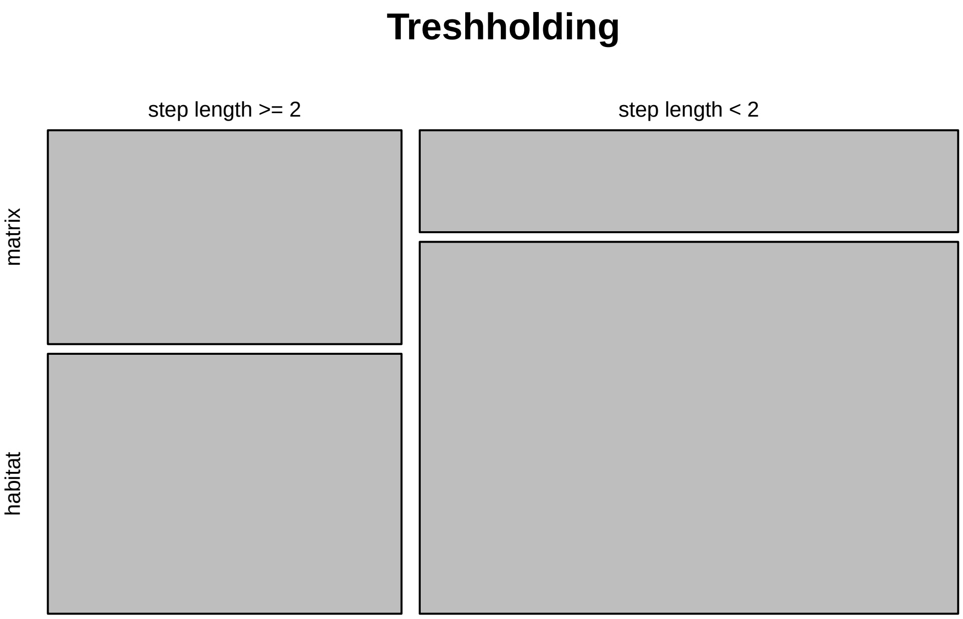

Finally, we can apply a Pearson's Chi-squared Test to determine if the observed proportions of each state within the habitat components are signficantly different from a random distribution.

```r
prop.table(tb, 1)
```

```
##
##                        matrix   habitat
##   step length >= 2 0.4513869 0.5486131
##   step length < 2  0.2151899 0.7848101
```

```r
prop.table(tb, 2)
```

```
##
##                        matrix   habitat
##   step length >= 2 0.5794603 0.3146877
##   step length < 2  0.4205397 0.6853123
```

```r
chisq.test(tb)
```

```
##
##  Pearson's Chi-squared test with Yates' continuity correction
##
## data:  tb
## X-squared = 539.16, df = 1, p-value < 2.2e-16
```

The test points out that the observed pattern of the state proportions is significant.

Behavioural change point analysis

Next, we are interested in finding the points where the animal starts to migrate.

```
xy$Time <- 1:nrow(xy)
path_char <- bcpa::MakeTrack(xy$x, xy$y, xy$Time)
path_char <- bcpa::GetVT(path_char)
path_char$nsd <- xy$nsd[-(1:2)]

# run the bcpa
ws <- WindowSweep(path_char, "nsd", windowsize = 30, progress = FALSE)

plot(ws, type = "flat", clusterwidth = 24 * 7,
     xlab = "time", las = 1, ylab = "net squared displacement")
```

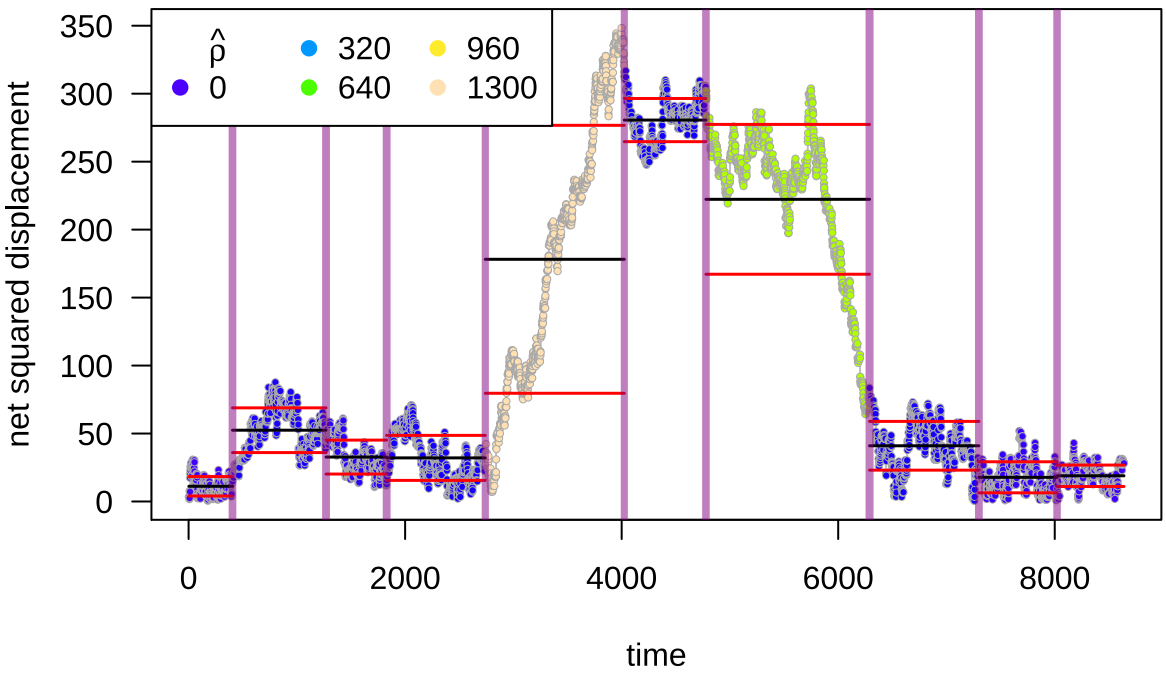


```
# add trajectory plot
xy_bc <- data.frame(Time=xy$Time, X=xy$x, Y=xy$y)
PathPlot(xy_bc,ws, type = "flat",clusterwidth = 24 * 7,
         plotlegend = TRUE, tauwhere = "topleft",
         n.legend = 4, ncol.legend = 2, bty.legend = TRUE)
```

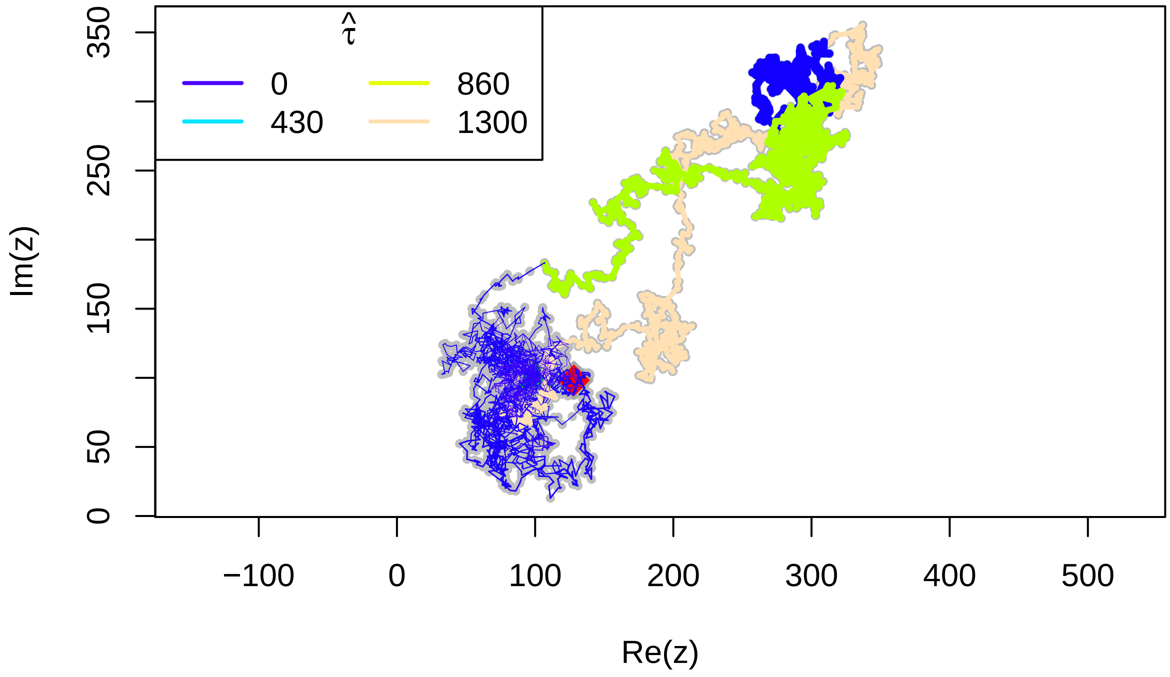

Hidden Markov Models

In the last step we want to find two different states of the animal (e.g., activ and resting) and model the transition probabilities as a function of habitat tand time of the day.

```
## moveHMM
mu0 <- c(0.1, 1)
sigma0 <- c(0.1, 1)
zeromass0 <- c(0.1, 0.05)
stepPar0 <- c(mu0, sigma0)
angleMean0 <- c(pi, 0)
kappa0 <- c(1, 1)
anglePar0 <- c(angleMean0, kappa0)
dat$hab <- factor(dat$hab)

m0 <- fitHMM(data = dat, nbStates = 2, stepPar0 = stepPar0, anglePar0 = anglePar0,
             formula = ~ 1)

m1 <- fitHMM(data = dat, nbStates = 2, stepPar0 = stepPar0, anglePar0 = anglePar0,
             formula = ~ hour + I(hour^2))

m2 <- fitHMM(data = dat, nbStates = 2, stepPar0 = stepPar0, anglePar0 = anglePar0,
             formula = ~ hour + I(hour^2) + hab)

AIC(m0, m1, m2)
```

```
##   Model      AIC
## 1    m2 52995.16
## 2    m1 53047.19
## 3    m0 54904.34
```

```r
# plots
z <- 1:24


plot(0, 0, type = "n", xlim = range(z), ylim = c(0, 1),
     xlab = "hour of day", ylab = "Probability to stay in state")
lines(z, 1 - plogis(m1$mle$beta[1, 1] +
                      m1$mle$beta[2, 1] * z + m1$mle$beta[3, 1] * z^2))
lines(z, 1 - plogis(m1$mle$beta[1, 2] +
                      m1$mle$beta[2, 2] * z + m1$mle$beta[3, 2] * z^2),
      col = "red")
```

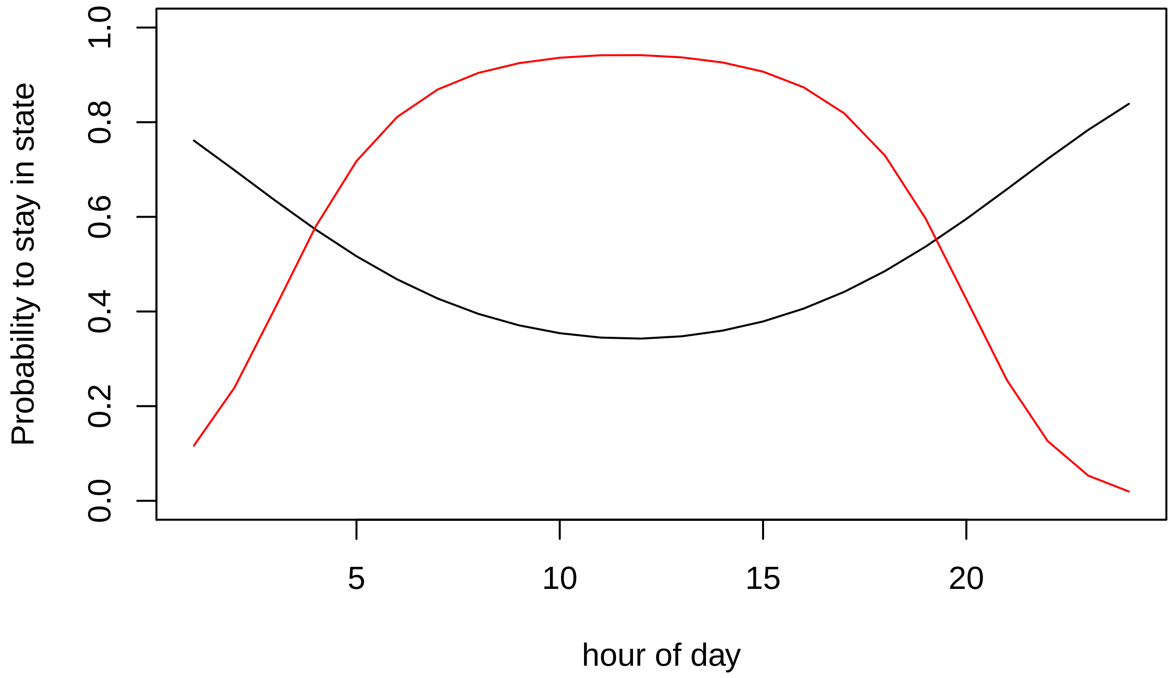


```r
plot(0, 0, type = "n", xlim = range(z), ylim = c(0, 1),
     xlab = "hour of day", ylab = "Probability to stay in state")
lines(z, plogis(m2$mle$beta[1, 1] + m2$mle$beta[2, 1] * z +
                  m2$mle$beta[3, 1] * z^2 + m2$mle$beta[4,1] * 1))
lines(z, plogis(m2$mle$beta[1, 1] + m2$mle$beta[2, 1] * z +
                  m2$mle$beta[3, 1] * z^2 + m2$mle$beta[4,1] * 0), lty = 2)

lines(z, plogis(m2$mle$beta[1, 2] + m2$mle$beta[2, 2] * z +
                  m2$mle$beta[3, 2] * z^2 + m2$mle$beta[4, 2] * 1),
      col = "red")
lines(z, plogis(m2$mle$beta[1, 2] + m2$mle$beta[2, 2] * z +
                  m2$mle$beta[3, 2] * z^2 + m2$mle$beta[4, 2] * 0),
      lty = 2, col = "red")

legend(17.5,0.25, c("inactive (habitat)",
                    "inactive (matrix)",
                    "active (habitat)", "active (matrix)"),
       lty = c(1,2,1,2), lwd = c(2,2,2,2), col=c("black","black", "red","red"))
```

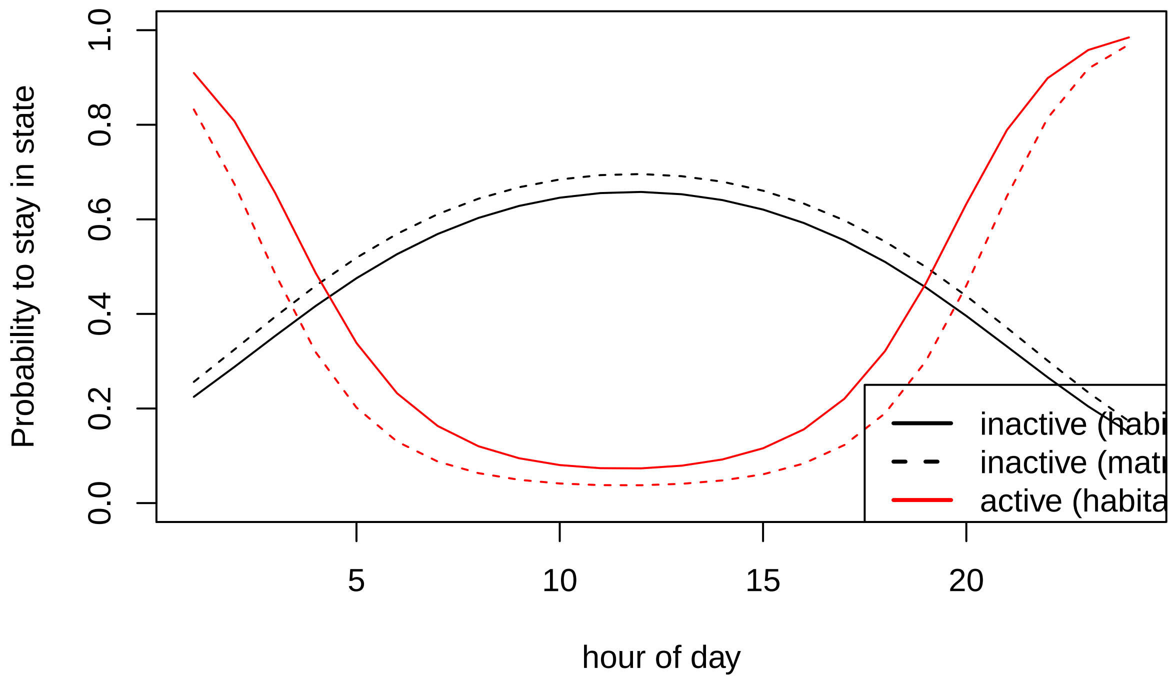

Probability to stay in state
0.0
0.2
0.4
0.6
0.8
1.0
5
10
15
20
hour of day
inactive (habi
inactive (matr
active (habita
